# Proteases and oxidant stress control organic dust induction of inflammatory gene expression in lung epithelial cells

**DOI:** 10.1186/s12931-016-0455-z

**Published:** 2016-10-22

**Authors:** Kartiga Natarajan, Koteswara R. Gottipati, Kiflu Berhane, Buka Samten, Usha Pendurthi, Vijay Boggaram

**Affiliations:** 1 Department of Cellular and Molecular Biology, University of Texas Health Science Center at Tyler, 11937 US Highway 271, Tyler, TX 75708-3154 USA; 2 Department of Pulmonary Immunology, University of Texas Health Science Center at Tyler, Tyler, TX USA

**Keywords:** Lung epithelial cells, Protease, Protease-activated receptors, Oxidant stress, Gene expression

## Abstract

**Background:**

Persistant inflammatory responses to infectious agents and other components in organic dust underlie lung injury and development of respiratory diseases. Organic dust components responsible for eliciting inflammation and the mechanisms by which they cause lung inflammation are not fully understood. We studied the mechanisms by which protease activities in poultry dust extracts and intracellular oxidant stress induce inflammatory gene expression in A549 and Beas2B lung epithelial cells.

**Methods:**

The effects of dust extracts on inflammatory gene expression were analyzed by quantitative polymerase chain reaction (qPCR), enzyme linked immunosorbent (ELISA) and western blot assays. Oxidant stress was probed by dihydroethidium (DHE) labeling, and immunostaining for 4-hydroxynonenal (4-HNE). Effects on interleukin-8 (IL-8) promoter regulation were determined by transient transfection assay.

**Results:**

Dust extracts contained trypsin and elastase activities, and activated protease activated receptor (PAR)-1 and -2. Serine protease inhibitors and PAR-1 or PAR-2 knockdown suppressed inflammatory gene induction. Dust extract induction of IL-8 gene expression was associated with increased DHE-fluorescence and 4-HNE staining, and antioxidants suppressed inflammatory gene induction. Protease inhibitors and antioxidants suppressed protein kinase C and NF-κB activation and induction of IL-8 promoter activity in cells exposed to dust extract.

**Conclusions:**

Our studies demonstrate that proteases and intracellular oxidants control organic dust induction of inflammatory gene expression in lung epithelial cells. Targeting proteases and oxidant stress may serve as novel approaches for the treatment of organic dust induced lung diseases. This is the first report on the involvement of oxidant stress in the induction of inflammatory gene expression by organic dust.

**Electronic supplementary material:**

The online version of this article (doi:10.1186/s12931-016-0455-z) contains supplementary material, which is available to authorized users.

## Background

Lung diseases in agricultural workers are one of the earliest recognized occupational hazards [1]. Workers in animal confinement buildings are at risk of developing respiratory symptoms and respiratory diseases as a result of exposure to airborne dust [2]. As a result of high-density animal farming in modern animal production facilities, known as concentrated animal feeding operations (CAFOs), workers are exposed to high levels of airborne dust [3]. Respiratory symptoms in agriculture workers are associated with increased levels of inflammatory cytokines, neutrophils and macrophages in the respiratory tract [4, 5]. Air in poultry CAFOs contains higher levels of dust and its constituents such as endotoxin, ammonia, carbon dioxide as well as bacteria and fungi compared to swine CAFOs [6]. Perhaps due to the presence of higher levels of dust in the poultry CAFOs, workers experience higher prevalence and severity of respiratory symptoms and chronic bronchitis compared to other agricultural workers [6–8]. Allergic and non-allergic rhinitis, hypersensitivity pneumonitis and occupational asthma are commonly found in poultry workers [8–10].

The respiratory epithelium in addition to serving as a physical barrier against particulates and microbial pathogens, also functions to regulate immune and inflammatory responses to control host defense. Lung epithelial cells produce chemokines, cytokines and other bioactive molecules in response to environmental agents to modulate host inflammatory and immune responses [11–13]. We found previously that lung epithelial and THP-1 cells express high levels of interleukin-8 (IL-8) in response to poultry dust extract treatment, and protein kinase signaling and transcriptional mechanisms mediate IL-8 induction [14]. Our studies also demonstrated that polymyxin B did not block induction of IL-8 expression indicating that endotoxin in dust extract may not act independently as an inducing agent [14]. Our studies on the effects of poultry dust extract on gene expression profiling showed that several cytokine, chemokine and inflammatory proteins are commonly up-regulated in lung epithelial and THP-1 cells [15]. The inductive effects on inflammatory gene expression in A549 and Beas2B cells were similar to effects in primary human small airway epithelial cells [15] indicating that the effects are independent of the malignant, transformed or normal origin of the cells. Further, we found similar inductive effects on inflammatory gene expression in lungs of mice exposed to dust extract validating the effects observed in vitro [15]. Components of organic dust responsible for the induction of inflammatory cytokine production and inflammatory responses have not been fully characterized. Identification of dust components that elicit inflammatory responses is necessary for the understanding of cellular and molecular mechanisms mediating dust induced inflammatory responses. Despite the higher prevalence and severity of respiratory symptoms and respiratory diseases in agricultural workers, our knowledge on the cellular and molecular mechanisms mediating organic dust induced lung inflammatory responses remains incompletely understood.

Much of our knowledge on the mechanisms underlying organic dust induced lung inflammatory responses has been obtained from studies of the effects of swine CAFO dust. Studies using organic dusts obtained from sources other than swine CAFOs are necessary to determine whether similar cellular and molecular mechanisms mediate lung inflammatory responses. We studied the effects of poultry dust considering the potential impact exposure to poultry environment may have on the respiratory health of the workers. Poultry production has increased rapidly in the United States and other countries outpacing pork and beef production and is a major economic driver in the agricultural sector. As a consequence of the rapid expansion of the poultry production industry, a very large number of workers are at risk of developing respiratory symptoms and diseases. The higher prevalence and severity of respiratory symptoms in poultry workers highlights differences between poultry and other agricultural exposures and could stem from higher levels of dust and its components as well as differences in the components per se. Because poultry dust contains higher levels of endotoxin, ammonia, and microbes that are different from other organic dusts, cellular and molecular mechanisms of lung inflammatory responses could be different from those elicited by other organic dusts. Information on cellular and molecular mechanisms underlying poultry dust induced lung inflammation and lung diseases is lacking. In particular, the involvement of proteases and intracellular oxidants in the control of induction of inflammatory gene expression in lung epithelial cells has not been studied previously.

In this study, we found that aqueous poultry dust extracts (hereafter referred to as dust extracts) contained trypsin and elastase-like protease activities, and inhibition of protease activities suppressed protein kinase C and NF-κB activation, and induction of inflammatory gene expression in lung epithelial cells. We also found that poultry dust extracts activated protease activated receptor (PAR)-1 and -2 to induce IL-8 expression. Exposure to poultry dust extracts increased reactive oxygen species (ROS) levels, and antioxidants suppressed protein kinase C and NF-κB activation and induction of inflammatory gene expression.

## Methods

### Preparation of dust extract

Settled broiler poultry dust had previously been collected from a commercial poultry farm in East Texas, USA. Dust was extracted at a ratio of 1:10 (wt/vol) with serum-free F12 K medium containing penicillin (100 U/ml), streptomycin (100 μg/ml) and amphotericin B (0.25 μg/ml) as described previously [14]. The concentration of this extract was arbitrarily considered as 100 %. The protein concentration of the dust extract was typically found to be in the range of 0.2–0.4 mg/ml.

### Chemicals

α1-antitrypsin, trypsin inhibitor from Glycine max (soybean), leupeptin, aprotinin were from Sigma-Aldrich, and dissolved in F12K medium without serum. GM6001 and E64 were from Santa Cruz Biotechnology, and dissolved in DMSO. Protease inhibitor cocktail solution in DMSO was from Sigma. It contained AEBSF (104 mM), aprotinin (80 μM), bestatin (4 mM), E64 (1.4 mM), leupeptin (2 mM) and pepstatin (1.5 mM). BMS200261 was from KeraFast (Boston, USA), and dissolved in water. N-acetylcysteine was from Sigma, and dissolved in cell culture medium without serum and pH adjusted to 7.0 with sodium hydroxide. Dimethylthiourea was from Acros, and dissolved in cell culture medium without serum. 1-(2-Cyano-3,12,28-trioxooleana-1,9(11)-dien-28-yl)-1*H*-imidazole (CDDOIm) was from the National Cancer Institute or Tocris, and dissolved in DMSO.

### Determination of trypsin and elastase activities

Trypsin and elastase activities in poultry dust extracts were determined using chromogenic p-nitroanilide substrates, Na-Benzoyl-D,L-arginine 4-nitroanilide hydrochloride (BAPNA) (Sigma) and N-Suc-Ala-Ala-Ala-pNA (SAPNA) (Elastin Products, Owensville, MO), respectively. For measurement of trypsin activity, dust extract was incubated with 0.46 mM BAPNA in 0.1 M Tris-HCl, pH 8.0 and for elastase activity, it was incubated with 0.375 mM SAPNA in 0.1 M Tris-HCl, pH 8.3 at room temperature and absorbance at 410 nm was measured at various times. Absorbance obtained with a blank reaction without dust extract was subtracted from absorbance obtained with dust samples. All measurements were done in duplicate.

### Cell culture

A549 (ATCC CCL185) lung epithelial cells were grown on plastic culture dishes in F12 K medium containing 10 % fetal bovine serum and penicillin (100 U/ml), streptomycin (100 μg/ml), and amphotericin B (0.25 μg/ml). Beas2B (ATCC TIB-202) bronchial epithelial cells were grown on plastic culture dishes coated with fibronectin, bovine type I collagen, and bovine serum albumin and maintained in LHC 9 medium (Invitrogen) containing penicillin (100 U/ml), streptomycin (100 μg/ml), and amphotericin B (0.25 μg/ml). Normal human small airway epithelial cells (SAEC) were purchased from Lonza and grown on plastic cell culture dishes in small airway growth medium (SAGM) (Lonza). Cells were placed in serum-free or basal medium overnight and then subjected to treatments.

### RNA isolation and real time quantitative RT-PCR

Total RNA was isolated using Tri-Reagent (Molecular Research Center), and genomic DNA digested by treatment with DNAse (Turbo DNA-free kit, Ambion). After genomic DNA digestion, RNA was quantified by measuring absorbance at 260 nm and cDNA synthesized using iScript Reverse Transcription kit (Bio-Rad). Levels of mRNAs and 18 S rRNA were determined by TaqMan probe based assay (Bio-Rad) using reaction conditions of 40 cycles of 95 °C for 30 s, 95 °C for 5 s and 60 °C for 30 s. Levels of mRNAs were normalized to 18 S rRNA levels. TaqMan gene expression IDs for target mRNAs are listed in Table 1.

**Table 1:** TaqMan gene expression IDs for genes quantified by real time quantitative RT-PCR

Gene symbol	Gene name	Human assay ID
IL-1β	Interleukin-1beta	Hs01555410_m1
IL-6	Interleukin-6	Hs00985639_m1
IL-8	Interleukin-8	Hs00174103_m1
ICAM-1	Intercellular adhesion molecule-1	Hs00164932_m1
CCL2	Chemokine (C-C motif) Ligand 2	Hs00234140_m1
TLR4	Toll-like receptor-4	Hs00152939_m1
PTGS2	Prostaglandin-endoperoxide synthase 2 (prostaglandin G/H synthase and cyclooxygenase)	Hs00153133_m1
F2R (PAR-1)	Thrombin receptor (PAR-1)	Hs00169258_m1
F2RL1 (PAR-2)	Coagulation factor II (thrombin) receptor-like 1 (PAR-2)	Hs00608346_m1
MMP-1	Matrix metalloproteinase-1	Hs00899658_m1
MMP-3	Matrix metalloproteinase-3	Hs00968305_m1
MMP-9	Matrix metalloproteinase-9	Hs00234579_m1
MMP-13	Matrix metalloproteinase-13	Hs00233992_m1
18 S	18 S ribosomal RNA	Hs99999901_s1

### ELISA

Cells were suspended in M-PER protein extraction reagent (Pierce) containing 150 mM NaCl and protease inhibitor cocktail (0.5 ×) and subjected to freeze-thaw for lysis. Cell lysates were centrifuged at ~ 16,000 g for 15 min at 4 °C and supernatants saved. IL-8 protein levels in cell medium and in cell lysate were quantified by ELISA (R & D) according to the manufacturer’s protocol.

### Dihydroethidium labeling of cells

A549 cells were grown on Permanox coverslips and Beas2B cells were grown on Permanox coverslips coated with fibronectin, bovine type I collagen and bovine serum albumin. Cells were maintained in serum-free medium overnight and incubated in the presence of 10 μM dihydroethidium (Sigma) for 1 h in the dark, rinsed and then exposed to medium alone or medium containing dust extract for 10 min. After exposure, coverslips were rinsed with cold phosphate-buffered saline, air-dried and mounted with Vectashield. Images were captured with a Nikon Eclipse TE2000-5 inverted fluorescent microscope equipped with an Ultra-VIEW LCI scanning confocal system (PerkinElmer Life Sciences) using 488 nm excitation and 568 nm emission filters. Imaging Suite version 5.0 acquisition and processing software was used to acquire the images.

### Immunostaining

Cells grown on coverslips were first subjected to antigen retrieval by heating in 10 mM sodium citrate, pH 6.0 containing 0.05 % Tween 20 at 95 °C for 5 min and then immunostained using Ultravision Detection System kit (Thermo Scientific) according to the kit instructions. Monoclonal mouse antibody against KLH-coupled 4-hydroxynonenal (R & D systems) was used at 5 μg/ml for immunostaining.

### Cell transfection and luciferase reporter assay

Control, thrombin R (PAR-1), and PAR-2 siRNAs (Santa Cruz Biotechnology) at 50 nM were transfected into cells using Lipofectamine 2000 (Invitrogen) according to the manufacturer’s protocol. After transfection for 5 h, cells were grown for 48 h to achieve knockdown of target RNAs and then subjected to treatments. Thrombin R and PAR-2 siRNAs are a pool of 3 target-specific siRNAs. Cells were transiently transfected with pGL3luc(basic)vector containing human IL-8 promoter linked to luciferase reporter gene along with pcDNA3.1, a β-galactosidase expression plasmid using Lipofectamine 2000 (Invitrogen) as described previously [14]. Luciferase and β-galactosidase activities in cell lysates were measured by chemiluminescent assays (Promega, Madison, WI; Tropix, Bedford, MA). Luciferase activities were normalized to cotransfected β-galactosidase activity or protein content of cell lysate.

### Protease activated receptor cleavage assays

Adenovirus vectors expressing PAR-1 or PAR-2 cleavage reporter constructs under control of CMV promoter were generated by fusing cDNA encoding secreted human alkaline phosphatase (AP) to the N-terminus of PAR-1 or PAR-2. The constructions of pcDNA3.0 plasmid and adenovirus (pacAd5CMV) (Cell Biolabs) expressing PAR-1-AP and PAR-2-AP constructs have been reported previously [16, 17]. A549 cells were transduced with recombinant adenovirus expressing PAR1-AP or PAR2-AP at a multiplicity of infection (MOI) of 10 per cell. After 24 h, cells were treated with medium or dust extract under serum-free conditions for various times and alkaline phosphatase activity in cell medium measured by chemiluminescent assay (Phospha-Light System, Applied Biosystems). Background alkaline phosphatase activity in medium from cells not transduced with adenovirus was subtracted. It was also found that dust extracts contain alkaline phosphatase activity, and phosphatase activity of cell medium arising from cleavage of PARs was subtracted from corresponding phosphatase activity of dust extract.

### Western immunoblotting

Cell lysates were subjected to SDS-PAGE on 10 % Bis-Tris gels using MOPS running buffer, and proteins transferred by electroblotting on to PVDF membranes. Membranes were first incubated with polyclonal antibodies against phospho-PKC (pan) (Cell Signaling) or phospho-NF-κB p65 (ser536) (Cell Signaling) overnight at 4 °C and then with goat anti-rabbit alkaline phosphatase-conjugated secondary antibody for 1 h at room temperature. Protein bands were visualized, according to enhanced chemifluorescence detection method (Amersham). Membranes were reprobed with α-tubulin (Thermo Scientific), actin (Thermo Scientific) or NF-κB p65 (Santa Cruz) antibodies.

### Statistical analyses

Data are shown as means ± SD or SE. The levels of mRNA, protein, or promoter activity in control or dust extract treated samples were arbitrarily considered as 100, and statistical significance was evaluated by one sample *t*-test. One-tailed *p* values <0.05 were considered significant.

## Results

### Dust extract contains trypsin and elastase-like activities

**Fig. 1:**
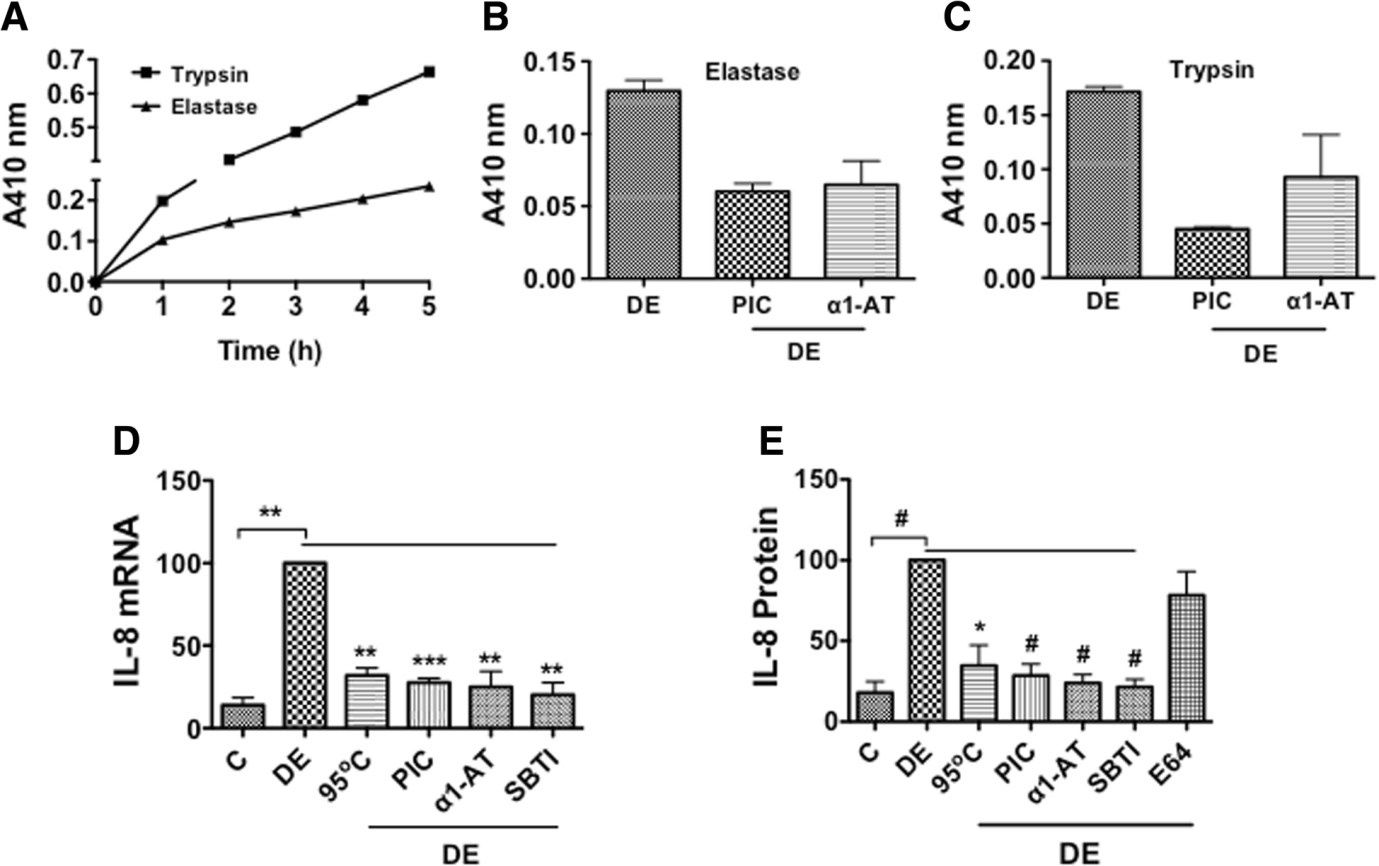
Protease activities in dust extract and the effects of protease inhibitors and heating on IL-8 mRNA and protein levels. **a** Trypsin and elastase activities in dust extract were measured using BAPNA and SAPNA substrates, respectively. Dust extract (5 μl) was mixed with BAPNA (0.92 mM) or SAPNA (0.37 mM) in a final volume of 200 μl of 0.1 M Tris-HCl 8.0 or 0.1 M Tris-HCl 8.3, incubated at room temperature and absorbance at 410 nm recorded at indicated times. Data shown are average of duplicate measurements. Similar results were obtained in a second independent experiment. **b** and **c** Trypsin and elastase activities were measured in the presence of protease inhibitor cocktail (0.5 ×) or α1-antitrypsin (10 μg) (α1-AT). Data shown are means ± SD of two independent experiments. **d** A549 cells were treated with medium (C), dust extract (0.25 %) (DE), dust extract (0.25 %) that was heated at 95 °C for 10 min, or dust extract (0.25 %) in the presence of 2 μl protease inhibitor cocktail (PIC), 10 μg/ml α1-antitrypsin (α1-AT), or 10 μg/ml soybean trypsin inhibitor (SBTI) for 3 h and IL-8 mRNA levels determined by qRT-PCR. IL-8 mRNA levels in dust extract treated cells were arbitrarily considered as 100, and relative IL-8 mRNA levels in other treatments are shown. Data shown are means ± SE (*n* = 3). **P < 0.01; ***P < 0.001. **e** A549 cells were treated with medium (C), dust extract (DE) (0.25 %), dust extract (0.25 %) heated at 95 °C for 10 min, or dust extract (0.25 %) in the presence of 25 μg/ml α1-antitrypsin (α1-AT), 25 μg/ml soybean trypsin inhibitor (SBTI), or 10 μM E64 for 3 h. IL-8 levels in cell medium were determined by ELISA. IL-8 levels in dust extract treated cells were arbitrarily considered as 100, and relative levels in other treatments are shown. Data are means ± SE (*n* = 3–6). *P < 0.05, ^#^ *P* < 0.0001

Poultry dust contains microbial pathogens, mites and animal dander, which could serve as potential sources for proteases. To determine if poultry dust contains proteases, we measured protease activities in aqueous dust extracts using chromogenic substrates for trypsin and elastase. Data showed that dust extracts displayed protease activity with BAPNA or SAPNA as a substrate that increased in a time-dependent manner indicating the presence of trypsin and elastase-like activities (Fig. 1a). Protease inhibitor cocktail and α1-antitrypsin suppressed elastase and trypsin activities confirming the presence of protease activities in dust extract (Fig. 1b, c).

### Heat and protease inhibitors suppress induction of IL-8 expression

To determine if protease activities control IL-8 expression, we tested the effects of heating, protease inhibitor cocktail and serine protease inhibitors such as α1-antitrypsin and soybean trypsin inhibitor on dust extract induction of IL-8 mRNA and IL-8 protein levels in A549 cells. The concentrations of α1-antitrypsin, leupeptin, aprotinin and E64 used in our experiments are similar to previously published studies [18–21]. Data showed that heating, protease inhibitor cocktail and serine protease inhibitors such as α1-antitrypsin and soybean trypsin inhibitor, but not E64, a cysteine protease inhibitor suppressed IL-8 mRNA and secreted IL-8 protein levels (Fig. 1d, e). IL-8 protein levels in A549 and Beas2B cell lysates and medium were similarly inhibited by several serine, but not cysteine protease inhibitors (Additional file 1: Figure S1A–D). Measurement of cell viability by MTS assay revealed that treatments with protease inhibitors did not adversely affect viability (Additional file 2: Figure S2A and C).

### α1-antitrypsin suppresses inflammatory gene induction

**Fig. 2:**
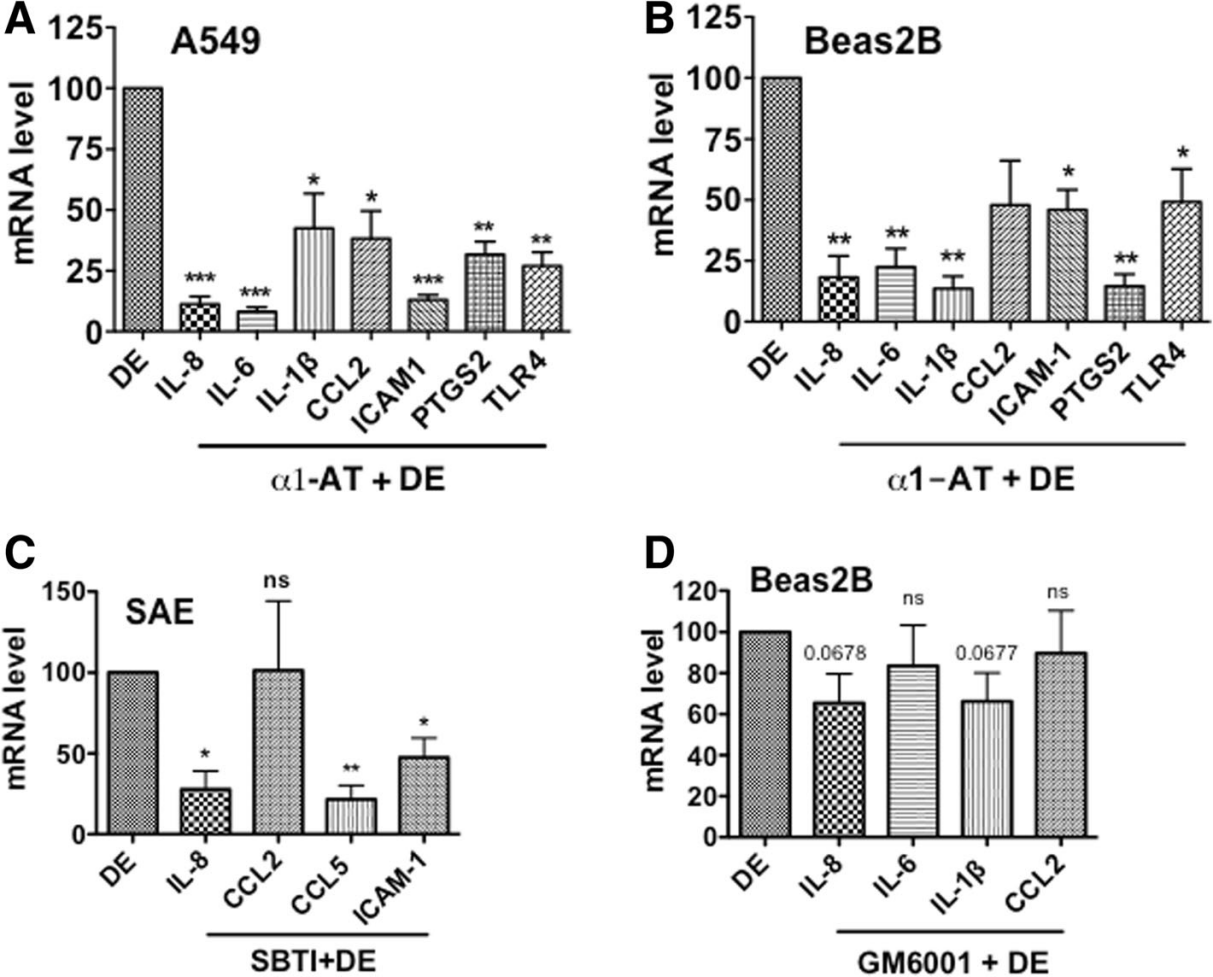
Effects of protease inhibitors on inflammatory gene induction. A549 (**a**), Beas2B (**b**) and SAE (**c**) cells were treated with medium, dust extract (DE) (0.25 %) alone or dust extract (0.25 %) in the presence of 25 μg/ml α1-antitrypsin (α1-AT) or 25 μg/ml soybean trypsin inhibitor for 3 h. Beas2B cells (**d**) were first treated with medium or medium containing metalloproteinase inhibitor GM6001 (10 μM) for 1 h and then exposed to medium or dust extract (DE) (0.25 %) for 3 h. Levels of mRNAs were determined by qRT-PCR. Levels of each mRNA in dust extract treated cells were arbitrarily considered as 100 and relative levels in cells treated with a combination of dust extract and inhibitors are shown. Data are means ± SE (*n* = 3). *P < 0.05; **P < 0.01; ***P < 0.001; ns, not significant, compared with cells treated only with dust extract

We found that serine protease inhibitors suppressed dust extract induction of IL-8 mRNA and protein levels in A549 and Beas2B cells. We have found previously that poultry dust extract induces the expression of cytokines, chemokines and other inflammatory proteins in A549, Beas2B and THP-1 cells [15]. To determine if proteases also control induction of other inflammatory genes, we investigated the effects of α1-antitrypsin or soybean trypsin inhibitor on the induction of IL-6, IL-1β, CCL2, CCL5, ICAM-1, PTGS2 and TLR4 mRNAs by dust extracts in A549, Beas2B and SAE cells. Results showed that α1-antitrypsin suppressed induction of these mRNAs by varying degrees in A549 and Beas2B cells. (Fig. 2a, b). In SAE cells, soybean trypsin inhibitor significantly inhibited dust extract induction of IL-8, CCL5 and ICAM-1 mRNAs, but had no effect on CCL2 mRNA. We did not investigate the effects on IL-6, IL-1β, PTGS2 and TLR4 mRNAs in SAE cells, as dust extracts did not significantly induce their levels [15]. Metalloproteinase inhibitor, GM6001 had modest inhibitory effects on the induction of IL-8 and IL-1β, but did not affect IL-6 and CCL2 mRNA levels (Fig. 2d). α1-antitrypsin and soybean trypsin inhibitor by themselves modestly increased (~2-fold) IL-6, IL-1β, CCL2, ICAM-1, PTGS2 and TLR4 mRNA levels, but had no effect on IL-8 mRNA levels (Additional file 3: Figure S3A and B).

### Dust extracts activate cleavage of protease-activated receptors

**Fig. 3:**
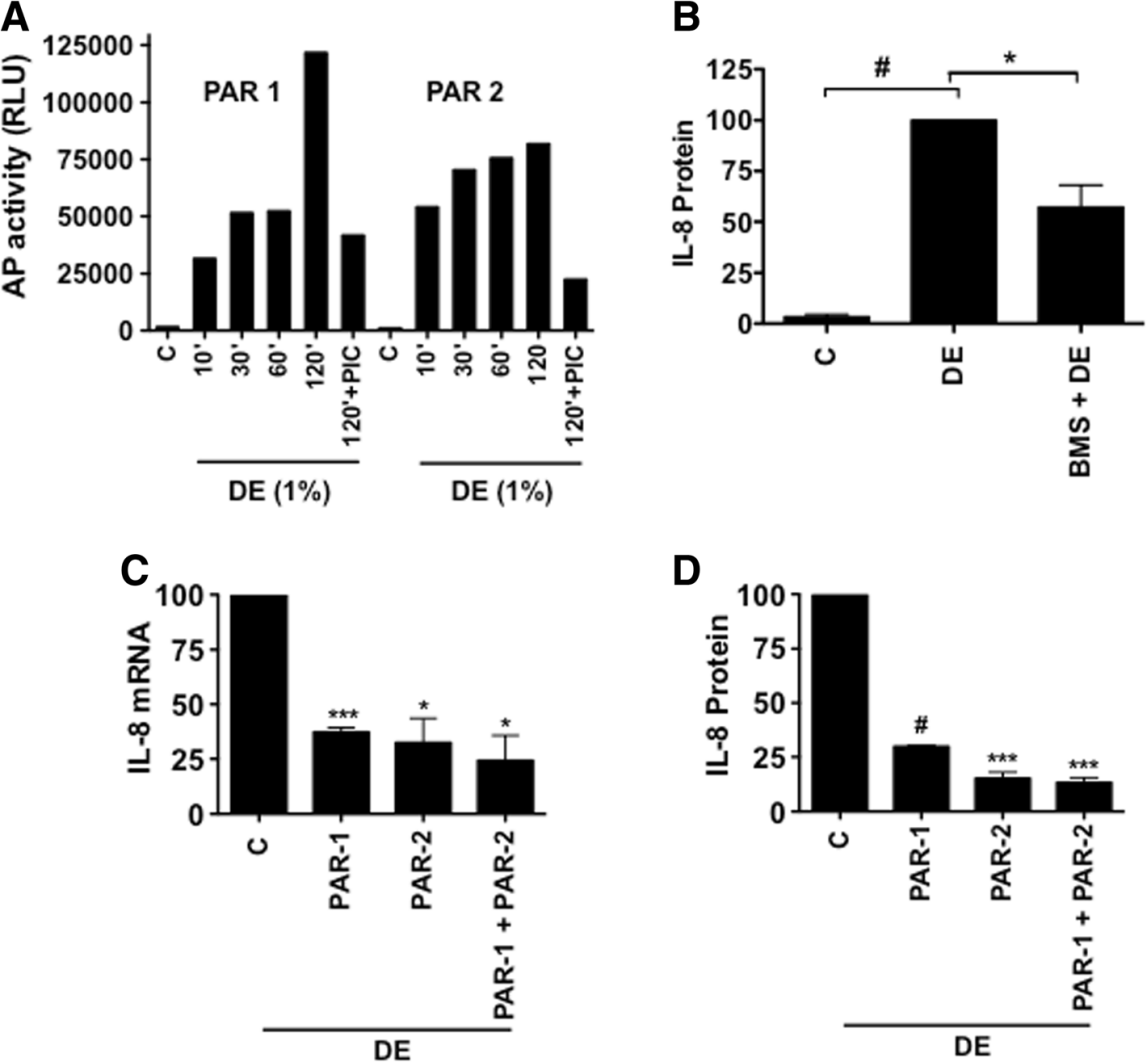
Activation of PAR-1 and -2, and the effects of PAR-1 antagonist and siRNA knockdown of PAR-1 and PAR-2 on the induction of IL-8 mRNA and IL-8 protein levels. **a** A549 cells were transduced with adenovirus expressing recombinant PAR-1 or PAR-2 containing alkaline phosphatase linked at the amino terminus, and treated with medium (C), dust extract (DE), or DE in the presence of protease inhibitor cocktail (PIC) and alkaline phosphatase activity in the medium was measured by chemiluminiscent assay. Data shown are means of duplicate measurements. Similar results were obtained in two other independent experiments. **b** A549 cells were first incubated with medium (C) or PAR-1 antagonist BMS200261 (50 μM) for 1 h and then treated with dust extract (DE) (0.25 %) for 3 h. IL-8 levels in the medium were measured by ELISA. Data shown are means ± SE (*n* = 4). *P < 0.05, #*P* < 0.0001. **c** and **d** A549 cells were transfected with control siRNA (C), PAR-1, PAR-2, or a combination of both siRNAs (PAR1 + PAR2) and after 60 h treated with medium or dust extract (0.25 %) (DE) for 3 h. IL-8 mRNA levels and secreted IL-8 protein levels in cell medium were determined by qRT-PCR and ELISA, respectively. IL-8 mRNA and protein levels in dust extract treated cells transfected with control siRNA (C) were arbitrarily set as 100 and relative levels in PAR-1 and PAR-2 siRNA transfected cells are shown. Data are means ± SE (*n* = 3). *P < 0.05; ***P < 0.001; #*P* < 0.0001 compared with cells transfected with control siRNA and treated with dust extract

Protease activated receptors (PARs) are important contributors of tissue inflammation in various diseases [22]. PARs 1–4 are expressed in the lung [23], and in particular, PAR-1 and PAR-2 have been implicated in airway inflammation and lung fibrosis [22, 24]. Furthermore, PAR-1 and -2 are activated by serine proteases such as trypsin and elastase [22]. To determine the involvement of PAR-1 and -2 in the induction of inflammatory gene expression by dust extract, we first investigated the effects of dust extracts on the activation of PAR-1 and -2. Recombinant adenovirus expressing PAR-1 or PAR-2 containing secreted alkaline phosphatase fused at the amino-terminus were expressed in A549 cells, and cells treated with dust extract alone or dust extract in the presence of protease inhibitor cocktail for the indicated times. Determination of alkaline phosphatase activity in cell culture medium demonstrated that dust extracts rapidly activated release of alkaline phosphatase in a time-dependent manner indicating cleavage of PAR-1 and -2 (Fig. 3a). Protease inhibitor cocktail significantly suppressed release of alkaline phosphatase activity further proving proteolytic cleavage of PAR-1 and -2 by dust extract (Fig. 3a).

### PAR-1 and PAR-2 activation is necessary for induction of IL-8

To determine the involvement of PAR-1 and PAR-2 in the induction of IL-8 expression by poultry dust extract, we first investigated the effects of PAR-1-specific antagonist BMS200261 [25] on IL-8 protein levels. Treatment with BMS200261 significantly suppressed induction of secreted IL-8 protein levels by dust extracts indicating that PAR-1 activation is necessary for IL-8 induction (Fig. 3b). Treatment with BMS200261, similarly suppressed cellular expression of IL-8 protein (C = 0.39 ± 0.08, DE = 100, BMS + DE = 40.85 ± 1.13, *n* = 2). To further prove the involvement of PAR receptors, we determined the effects of siRNA knockdown of PAR-1 and PAR-2 on the induction of IL-8 expression. Effects of dust extract on IL-8 mRNA and secreted IL-8 protein levels were determined in A549 cells after siRNA knockdown of PAR-1, PAR-2 or a combination of both and compared with IL-8 levels induced by dust extracts in cells transfected with control siRNA. Knockdown of PAR-1, PAR-2 or a combination of both suppressed dust extract induction of IL-8 mRNA and IL-8 protein levels by 70 % or greater compared with cells transfected with control siRNA (Fig. 3c, d). To ensure knockdown, we quantified PAR-1 and PAR-2 mRNA levels in PAR-1 or PAR-2 siRNA transfected cells and found greater than 80 % decrease in their levels (PAR-1 mRNA: control siRNA = 100, PAR-1 siRNA = 15.38 ± 8.37, *n* = 2; PAR-2 mRNA: control siRNA = 100, PAR-2 siRNA = 11.7 ± 7.49, *n* = 2).

### α1-antitrypsin and soybean trypsin inhibitor suppress induction of matrix metalloproteinases

**Fig. 4:**
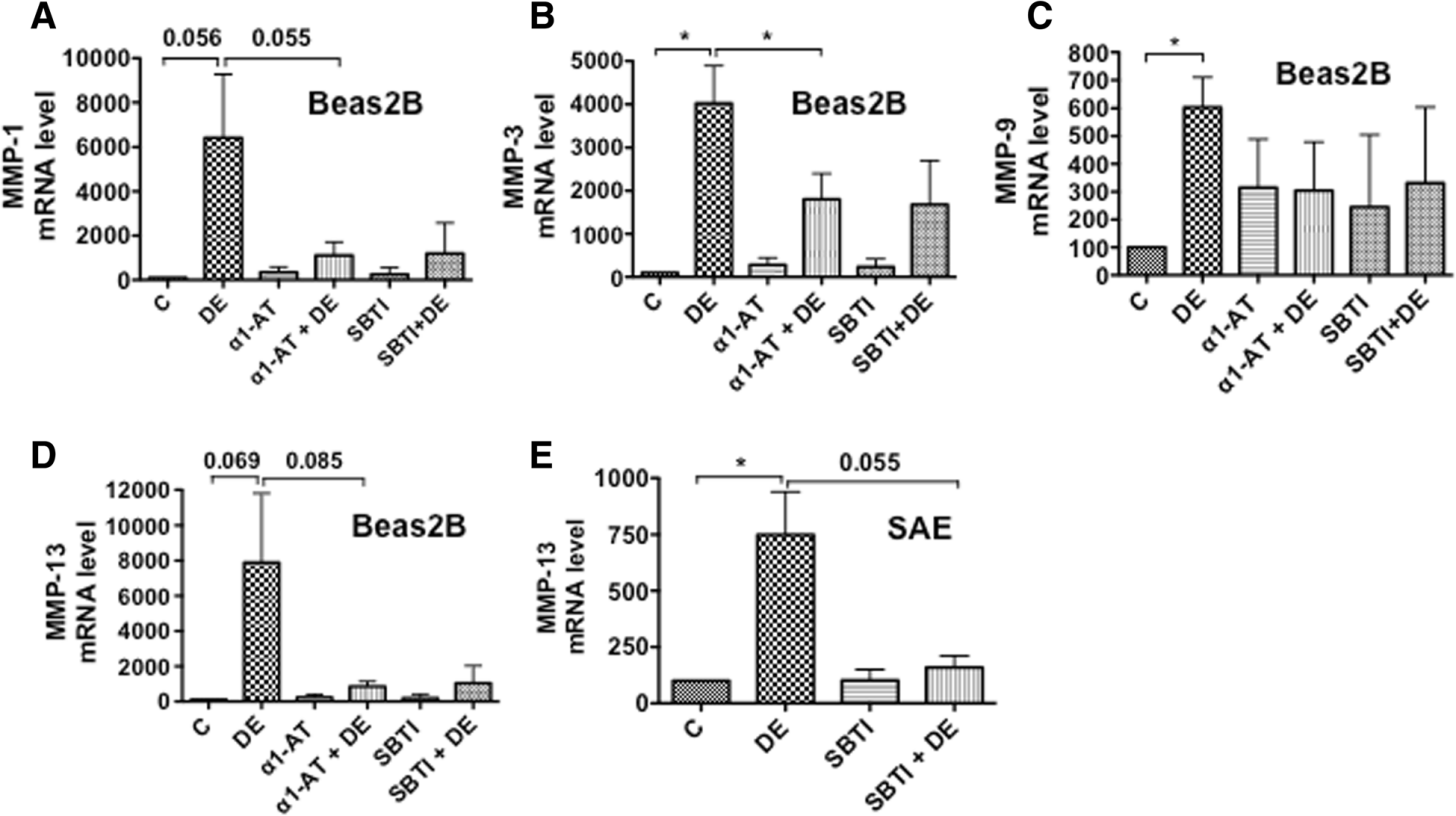
Effects of protease inhibitors on the induction of matrix metalloproteinases (MMPs) mRNA levels. **a**–**d** Beas2B cells (**a**–**d**) were treated with medium (C), dust extract (DE) (0.25 %), α1-antitrypsin (10 ug/ml) (α1-AT) alone, soybean trypsin inhibitor (10 μg/ml) (SBTI) alone or dust extract in the presence of α1-antitrypsin (10 μg/ml) or soybean trypsin inhibitor (10 μg/ml) for 3 h. SAE cells (**e**) were treated similarly with SBTI (10 ug/ml) and levels of MMP mRNAs were determined by qRT-PCR. The levels of each mRNA in control cells were arbitrarily considered as 100 and relative levels in other treatments are shown. Data shown for Beas2B cells are means ± SE (*n* = 3–4) for C, DE, α1-AT + DE samples and means ± SD (*n* = 2) for α1-AT, SBTI, SBTI + DE and for SAE cells are means ± SE (*n* = 3). *P < 0.05

Matrix metalloproteinases degrade extracellular matrix [26] and process cytokines and chemokines [27] to influence tissue remodeling and inflammatory responses. Recently, MMP-1 and MMP-13 were demonstrated to cleave and activate PAR-1 at noncanonical sites to elicit signaling patterns distinct from that seen with thrombin [28–30]. This suggests that MMP mediated cleavage and activation of PAR-1 could play important functions in the control of inflammatory gene expression. To determine if dust extracts induce expression of MMPs, particularly MMP-1 and MMP-13 that are known to activate PAR-1, we studied the effects of dust extracts on the levels of MMP mRNAs. We found that treatment of Beas2B cells with dust extracts increased the expression of MMP-1, MMP-3, MMP-9 and MMP-13 mRNA levels. The inductive effects on MMPs-1, -3 and -13 mRNA levels were more pronounced than on MMP-9 mRNA, and α1-antitrypsin and soybean trypsin inhibitor suppressed the induction of MMP mRNAs (Fig. 4a–d). In SAE cells, dust extracts significantly induced MMP-13 mRNA levels (Fig. 4e), but had no effect on the expression of MMP-1, MMP-3 and MMP-9 mRNAs (data not shown). As in the case of Beas2B cells, soybean trypsin inhibitor suppressed MMP-13 mRNA levels in SAE cells (Fig. 4e).

### Dust extract induces intracellular reactive oxygen species levels

**Fig. 5:**
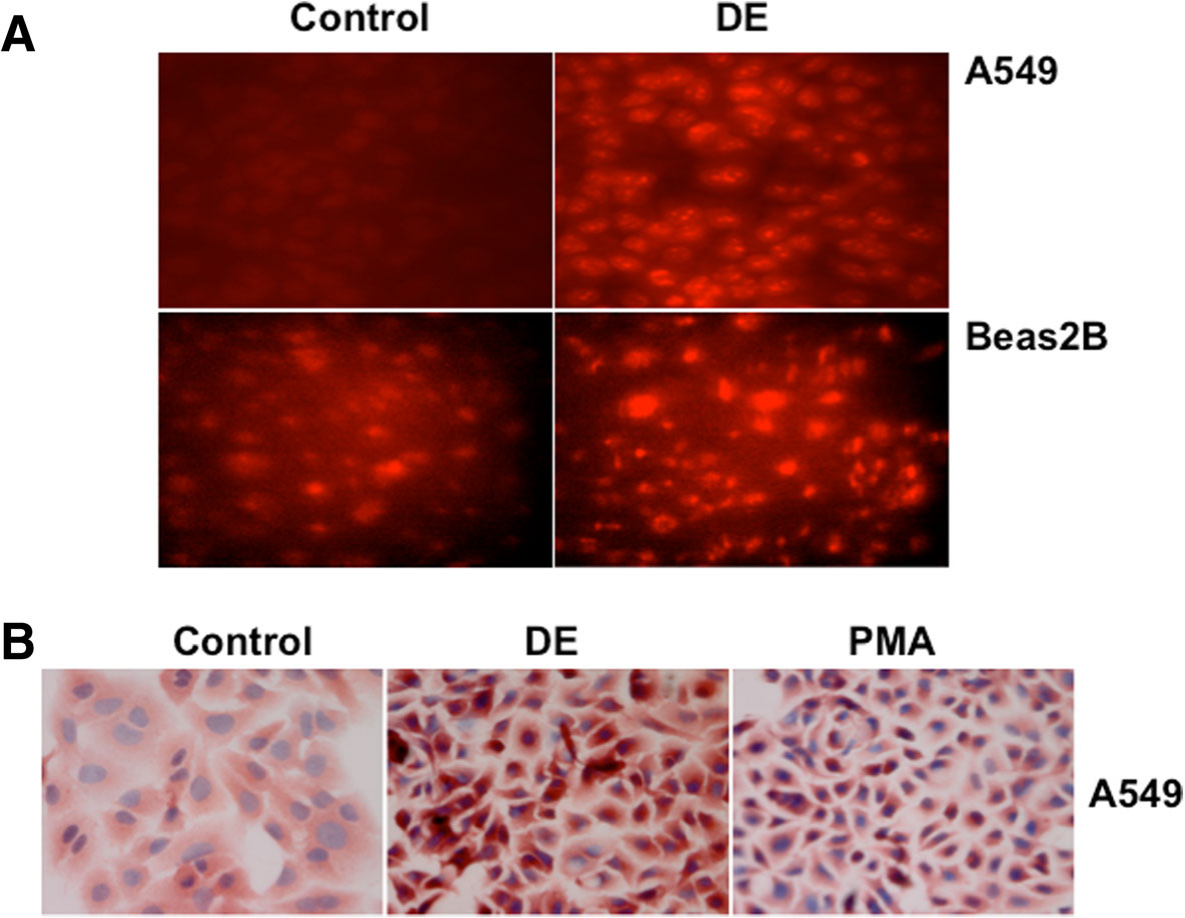
Dust extracts induce intracellular reactive oxygen species levels. **a** A549 and Beas2B cells grown on coverslips were incubated with 10 μM dihydroethidium for 1 h in the dark in serum-free medium and then exposed to medium alone (Control) or medium containing dust extract (1 %) (DE) for 10 min. After exposure, slides were viewed under a fluorescent microscope equipped with an Ultra-VIEW LCI scanning confocal system using 488 nm excitation and 568 nm emission filters. Fluorescent images are representative of three independent experiments. Red color indicates intracellular ROS production. **b** A549 cells grown on coverslips were treated with medium alone (Control), dust extract (DE) (1 %) or phorbol myristate acetate (PMA) (10 nM) for 1 h under serum-free conditions. Afterwards, hydroxynonenal conjugated proteins were visualized by immunostaining. Images shown are representative of two independent experiments. PMA was used as a positive control for the generation of intracellular reactive oxygen species (ROS). Magnification, 40×

Our studies have shown that poultry dust extract induces the expression of inflammatory cytokines, chemokines, and other inflammatory proteins in A549, Beas2B and THP-1 cells [15]. Because oxidant stress is linked to inflammation [31] and underlie respiratory diseases [32], we determined if dust extract treatment increases oxidant levels in A549 and Beas2B cells. We assessed the effects of dust extracts on the intracellular generation of reactive oxygen species (ROS) in A549 and Beas2B cells with dihydroethidium (DHE), a selective indicator of superoxide anion. We found that treatment with dust extracts increased DHE-derived fluorescence at 568 nm in A549 and Beas2B cells compared to untreated cells indicating increased production of intracellular superoxide anion (Fig. 5a). This was particularly evident in A549 cells, which displayed very low fluorescence under untreated conditions. On the other hand, untreated Beas2B cells displayed higher levels of fluorescence, which further increased upon treatment of cells with dust extract. Treatment of A549 cells with dust extracts increased immunostaining for 4-hydroxynonenal (4-HNE) (Fig. 5b), a marker of lipid peroxidation, further supporting the evidence for elevated intracellular oxidant levels. Cells treated with phorbol myristate acetate (PMA), known to increase intracellular oxidant levels, served as a positive control for 4-HNE immunostaining.

### Antioxidants suppress inflammatory gene expression

**Fig. 6:**
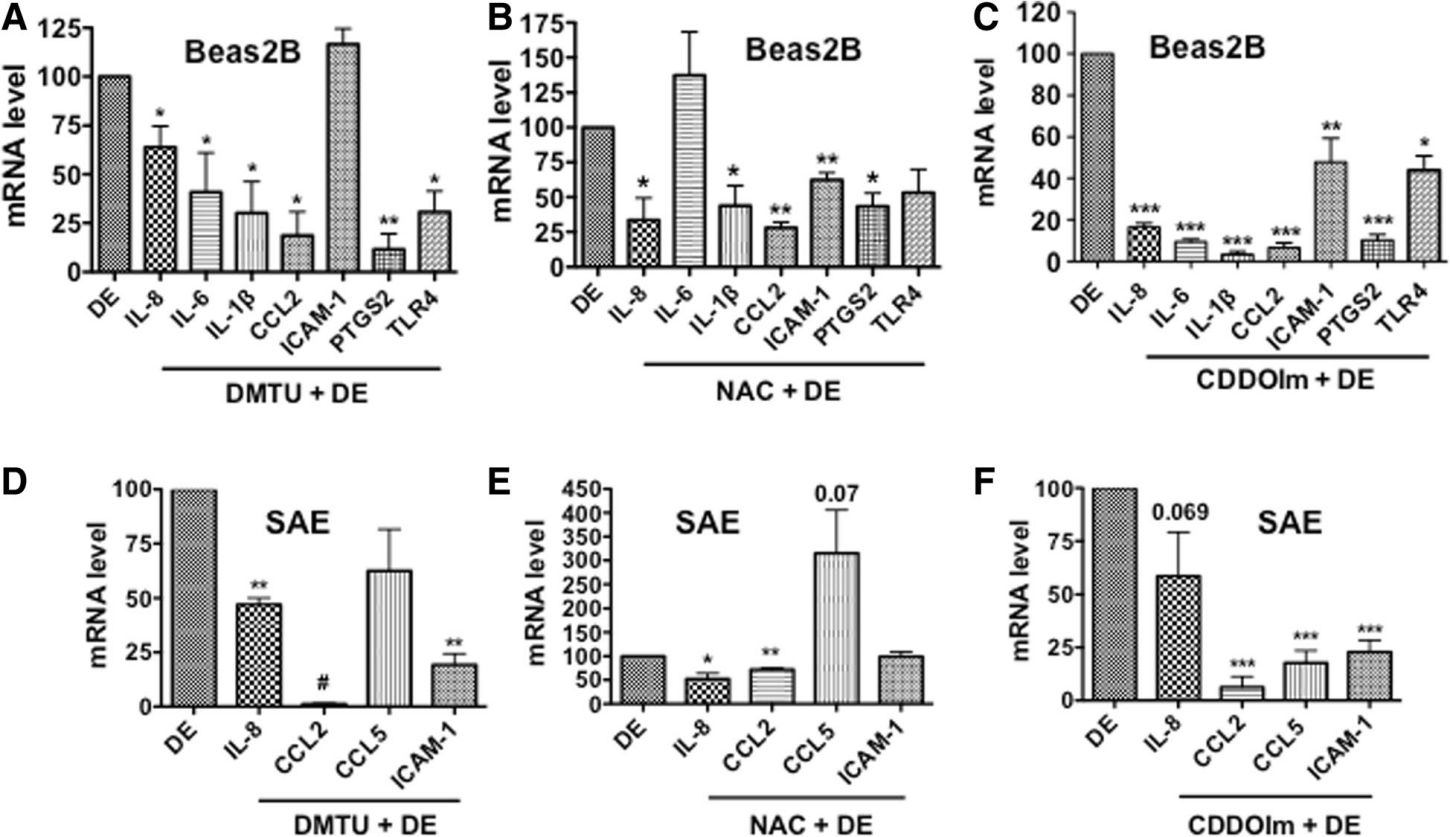
Effects of antioxidants on the induction of inflammatory gene mRNA levels. Beas2B (**a**–**c**) and SAE (**d**–**f**) cells were first incubated with medium or medium containing 30 mM DMTU for 1 h or 15 mM n-acetylcysteine (NAC) adjusted to pH 7 or 0.5 μM 1-(2-Cyano-3,12,28-trioxooleana-1,9(11)-dien-28-yl)-1H-imidazole (CDDOIm) for 3 h and then treated with medium or 0.25 % dust extract (DE) for 3 h. Levels of mRNAs were determined by qRT-PCR. Levels of mRNAs in dust extract treated cells were arbitrarily considered as 100, and relative levels in cells treated with a combination of antioxidant and dust extract are shown. Data shown are means ± SE (*n* = 3–4) except in the case of TLR4 for Beas2B cells treated with a combination of CDDOIm and DE, in which case data is shown as mean ± SD (*n* = 2). *P < 0.05; **P < 0.01; ***P < 0.001, #*P* < 0.0001 compared with cells treated with dust extract alone

To determine if oxidants generated due to dust extract exposure are involved in the induction of inflammatory gene expression, we first investigated the effects of various antioxidants on the induction of IL-8 mRNA and IL-8 protein levels in A549 and Beas2B cells (Additional file 4: Figure S4A–D). In particular, we tested the effects of dimethyl sulfoxide (DMSO), dimethylthiourea (DMTU), and mannitol which are known to scavenge hydroxyl radicals, diphenyleneiodonium (DPI), an inhibitor of NAPDH oxidase and n-acetylcysteine (NAC), which is known to elevate cellular reduced glutathione levels. As mannitol is impermeable to cells, it was used to distinguish the effects of intracellular versus extracellular oxidants on IL-8 induction. We found that in A549 cells, DMSO, DMTU, mannitol, or DPI had no effect on the induction of IL-8 mRNA levels, however n-acetylcysteine suppressed induction of IL-8 mRNA levels by approximately 50 % (Additional file 4: Figure S4A). In Beas2B cells, DMSO, DMTU, and NAC, but not mannitol suppressed IL-8 mRNA levels induced by poultry dust extract by approximately 50 %. Induction of IL-8 mRNA levels was not affected by DPI in Beas2B cells as in the case of A549 cells (Additional file 4: Figure S4C). The effects of various antioxidants on IL-8 protein levels in cell medium were similar to effects on IL-8 mRNA levels (Additional file 4: Figure S4B and D). Although DMTU did not suppress the induction of IL-8 mRNA levels in A549 cells, it suppressed the induction of IL-6 (dust extract = 100, DMTU + dust extract = 31.72 ± 12, *n* = 3) and CCL2 (dust extract = 100, DMTU + dust extract = 44.4 ± 16.6, *n* = 3) mRNAs. Because DMTU and NAC significantly suppressed IL-8 mRNA and IL-8 protein levels in Beas2B cells, we determined their effects on the expression of other inflammatory genes. In Beas2B cells, DMTU suppressed the induction of IL-8, IL-6, IL-1β, CCL2, PTGS2 and TLR4 mRNAs, but had no effect on ICAM-1 mRNA levels, while NAC suppressed IL-8, IL-1β, CCL2, PTGS2, ICAM-1 and TLR4 mRNAs but had no effect on IL-6 mRNA (Fig. 6a, b). In SAE cells, DMTU suppressed IL-8, CCL2 and ICAM-1 mRNA levels, but had modest inhibitory effects on CCL5 mRNA levels, while NAC modestly suppressed IL-8 mRNA levels, but had no effect on CCL2 and ICAM-1 mRNA levels (Fig. 6d, e). Interestingly, NAC induced CCL5 mRNA levels to a greater level than that induced by dust extract alone (Fig. 6e).

To further corroborate the involvement of oxidants, we determined the effects of 1-(2-Cyano-3,12,28-trioxooleana-1,9(11)-dien-28-yl)-1*H*-imidazole (CDDOIm), a potent inducer of the master antioxidant transcription factor, Nrf2 on dust extract induction of inflammatory gene expression in Beas2B and SAE cells (Fig. 6c, f). We found that CDDOIm suppressed the induction of IL-8, IL-6, IL-1β, CCL2, ICAM-1, PTGS2 and TLR4 mRNAs in Beas2B cells (Fig. 6c). In SAE cells, CDDOIm modestly suppressed IL-8 mRNA levels, but had significant inhibitory effects on CCL2, CCL5 and ICAM-1 mRNA levels (Fig. 6f). In A549 cells, CCDOIm suppressed IL-8, IL-6, IL-1β, CCL2 and ICAM-1 mRNA levels, but had no affect on PTGS2 and TLR4 mRNA levels (Additional file 4: Figure S4E). CDDOIm and DMTU did not significantly alter A549 or Beas2B cell viability (Additional file 2: Figure S2). DMTU by itself reduced IL-8, IL-6, IL-1β, CCL2 and TLR4 mRNA basal levels, while NAC reduced CCL2 and CDDOIm reduced IL-1β, and CCL2 mRNA basal levels (Additional file 3: Figure S3C–E). We determined the levels of some of the Nrf2 target genes to ensure that the effects of CDDOIm are elicited via Nrf2 activation, and found that CDDOIm induced mRNA levels of heme oxygenase-1 (HMOX-1) (3.81 ± 0.41, *n* = 2), NADPH quinone oxidoreductase 1 (NQO1) (1.9 ± 0.54, *n* = 2) and glutamate cysteine ligase catalytic subunit (GCLC) (2.94 ± 0.23, *n* = 2) compared to levels in untreated cells. These genes are known to play important roles in the maintenance of cellular redox status, in particular GCLC controls the first rate limiting reaction in the biosynthesis of glutathione. These data suggest that the suppressive effects of CDDOIm could be mediated via enhancement of cellular redox status.

### Antioxidants suppress induction of matrix metalloproteinases

**Fig. 7:**
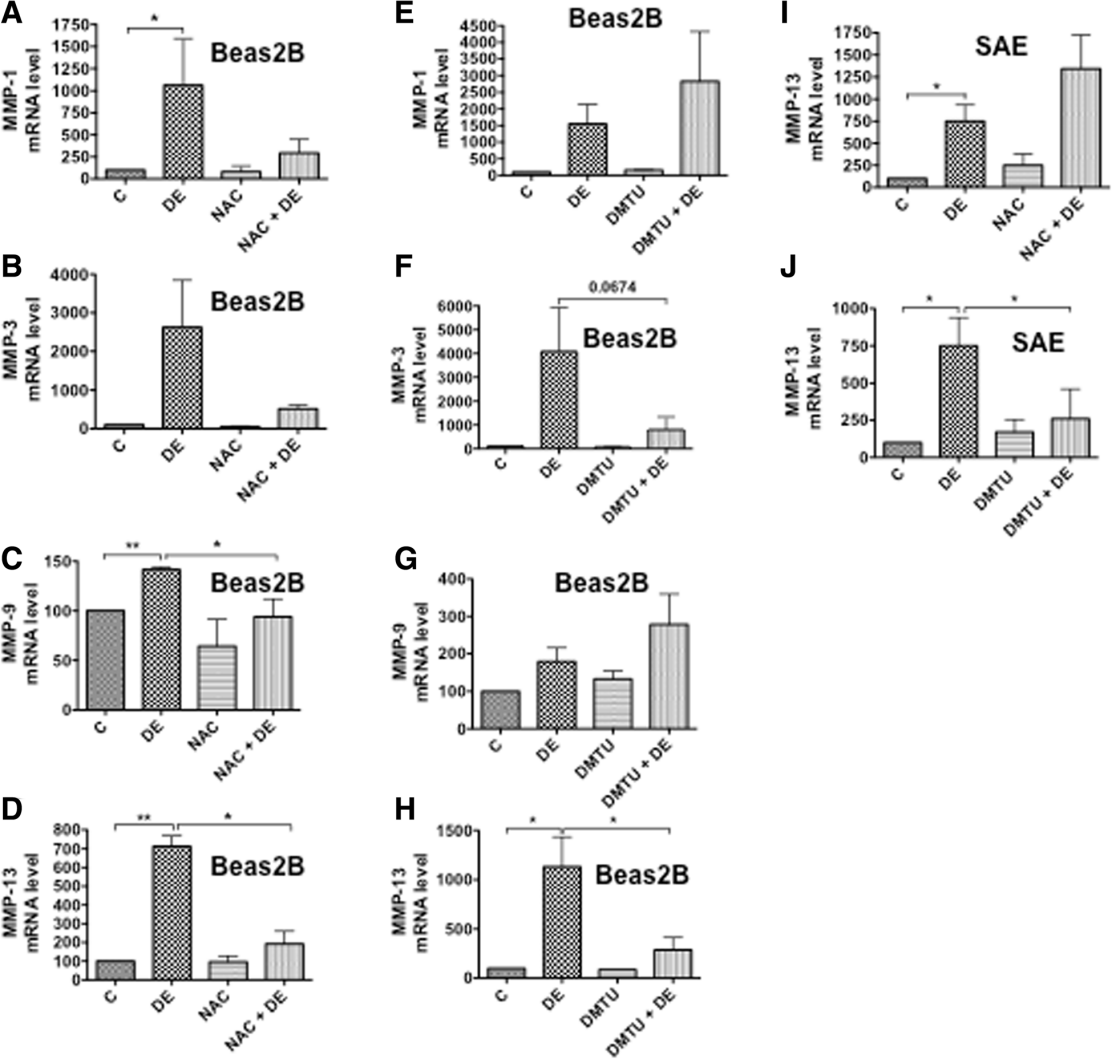
Effects of antioxidants on the induction of matrix metalloproteinase (MMP) mRNA levels. Beas2B (**a**–**h**) and SAE (**i** and **j**) cells were incubated first with medium (C), 15 mM n-acetylcysteine (NAC) adjusted to pH 7.0 for 3 h, or dimethylthiourea (DMTU) (30 mM) for 1 h and then treated with or without dust extract (0.25 %) (DE) for 3 h. Levels of MMP mRNAs were determined by qRT-PCR. Data shown are means ± SE (*n* = 3 for NAC and N = 3 for DMTU). Levels of each mRNA in control cells were arbitrarily considered as 100, and relative levels in treated cells are shown. *P < 0.05; **P < 0.01

We found that dust extract induces MMPs-1, -3, -9, and -13 mRNAs in Beas2B cells and MMP-13 mRNA in SAE cells. We investigated the effects of DMTU and n-acetylcysteine to understand the involvement of intracellular oxidants in the induction of MMP mRNA expression. N-acetylcysteine suppressed the induction of MMPs-1, -3, -9 and -13 although the effect on MMP-9 was less compared to the other MMPs (Fig. 7a–d). DMTU suppressed MMP-3 and MMP-13 but had modest inductive effects on MMP-1 and MMP-9 mRNAs in dust extract treated cells (Fig. 7e–h). In SAE cells, DMTU, but not NAC suppressed MMP-13 mRNA levels (Fig. 7i, j).

### α1-antitrypsin and CDDOIm suppress IL-8 promoter activity

**Fig. 8:**
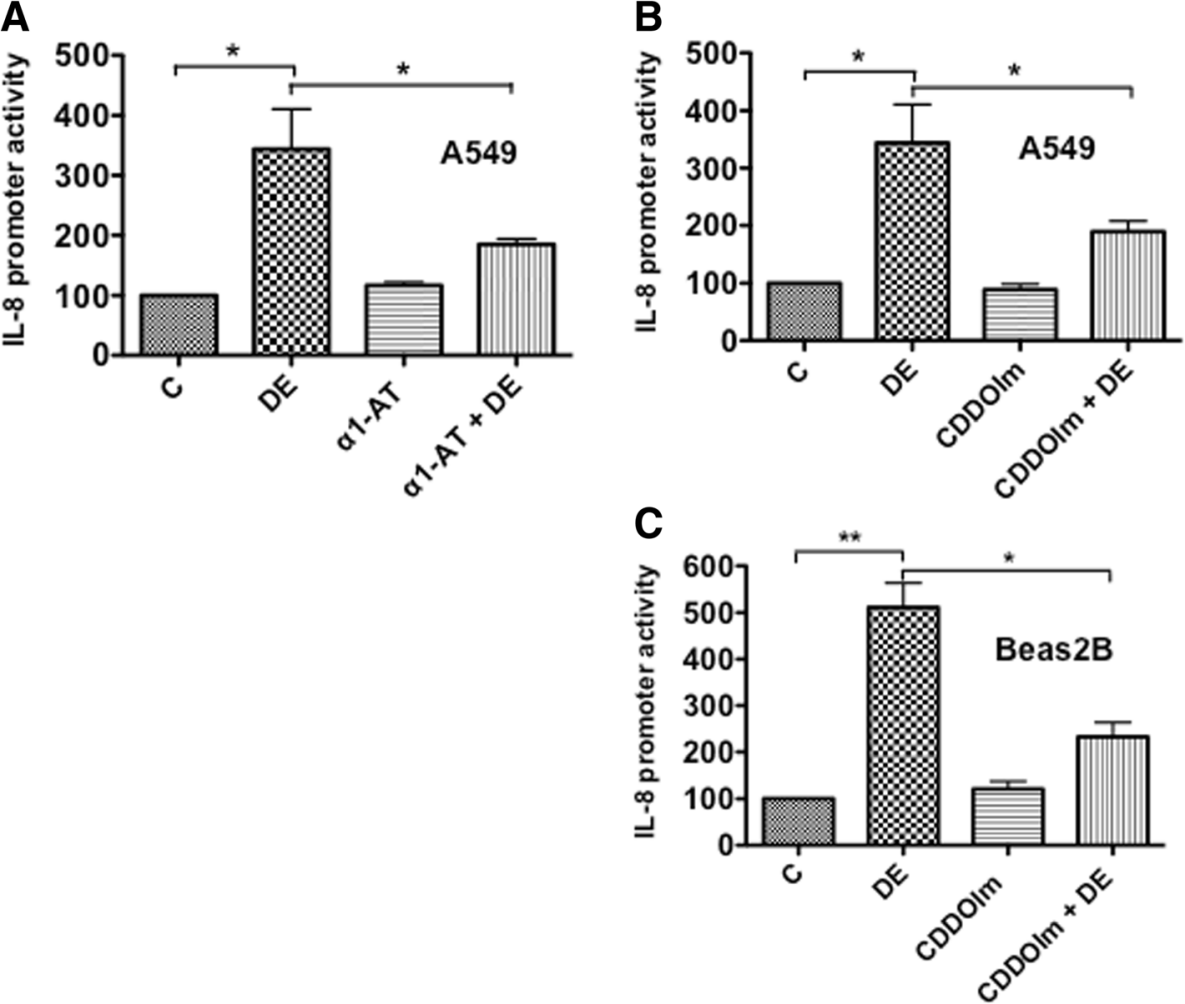
Effects of α1-antitrypsin and CDDOIm on the induction of IL-8 promoter activity. **a** A549 cells were transfected with an IL-8 promoter plasmid containing −144/+44 bp of human IL-8 promoter sequence linked to luciferase reporter gene. Transfected cells were treated with medium (C), 0.25 % dust extract (DE), 10 μg/ml α1-antitrypsin alone (α1-AT), or 0.25 % dust extract in the presence of 10 μg/ml α1-antitrypsin for 6 h. Luciferase activities in cell lysates were measured and normalized to total cell protein. Luciferase activity in untreated cells (C) was arbitrarily set as 100. Data shown are means ± SE (*n* = 4). *P < 0.05. **b** and **c** After transfection with an IL-8 promoter plasmid (−144/+44 bp), A549 (**b**) and Beas2B (**c**) cells were incubated first with medium (C) or 1-(2-Cyano-3,12,28-trioxooleana-1,9(11)-dien-28-yl)-1H-imidazole (CDDOIm) (1 μM for A549 and 0.5 μM for Beas2B) for 3 h and then treated with or without dust extract (0.25 %) (DE) for 6 h. Luciferase activities were normalized to total cell protein. Data shown are means ± SE (*n* = −3). *P < 0.05 and **P < 0.01

Our studies showed that proteases in poultry dust extract and intracellular oxidants generated upon exposure of cells to poultry dust extract induce expression of IL-8 and other inflammatory genes. To understand mechanisms underlying the inductive effects of proteases and oxidants, we investigated the effects of α1-antitrypsin and CDDOIm on IL-8 promoter activity in A549 and Beas2B cells (Fig. 8a–c). We found that α1-antitrypsin and CDDOIm suppressed induction of IL-8 promoter activity by poultry dust extract indicating that transcriptional mechanisms mediate protease and oxidant induction of IL-8 expression.

### Soybean trypsin inhibitor and antioxidants suppress NF-κB activation

**Fig. 9:**
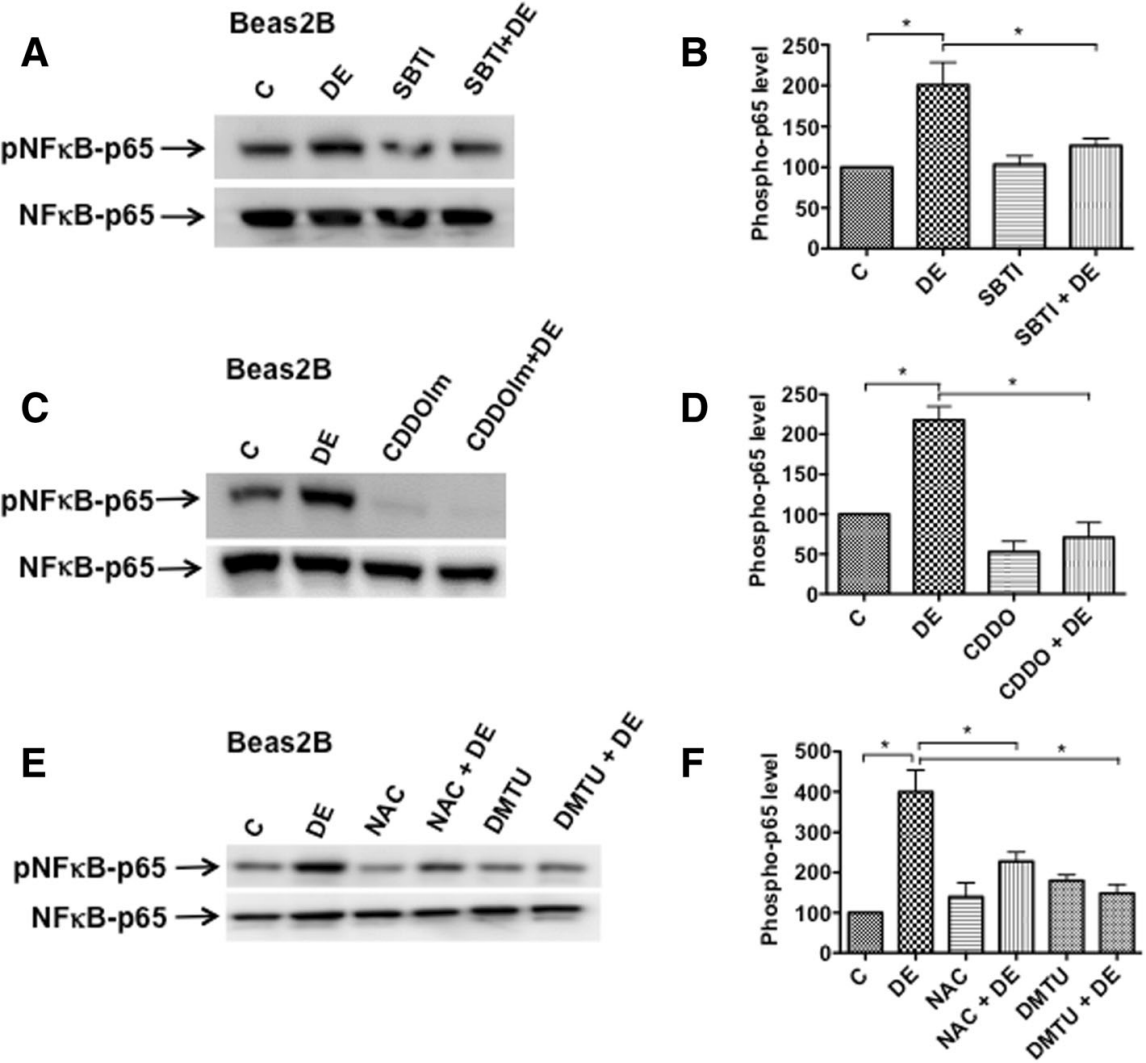
Effects of protease inhibitor and antioxidants on the activation of NFκB-p65. **a** Beas2B cells were treated with medium (C), 1 % dust extract (DE), 25 μg/ml soybean trypsin inhibitor (SBTI), or a combination of DE and SBTI for 5 min. A representative Western blot depicting the levels of phosphorylated NFκB-p65 and total NFκB-p65 is shown. **c** and **e** Beas2B cells were incubated first with medium (C), 0.5 μM CDDOIm or 15 mM n-acetylcysteine (NAC) adjusted to pH 7.0 for 3 h or 30 mM dimethylthiourea (DMTU) for 1 h and then treated with or without 1 % dust extract for 5 min. **b**, **d** and **f** The levels of phosphorylated NFκB-p65 were normalized to total NFκB-p65 and the levels in cells treated with medium (C) were arbitrarily considered as 100 and relative levels in treated cells are shown. Data shown are means ± SE (*n* = 3). * *P* < 0.05

We previously showed that poultry dust extracts activate NF-κB to induce IL-8 promoter activity in A549 and Beas2B cells [14]. We investigated whether or not proteases in dust extracts and intracellular oxidant generation are involved in the activation of NF-κB by analyzing effects on the phosphorylation of NFκB-p65. Phosphorylation status of NFκB-p65 is known to control transcriptional activation of NFκB-p65 [33, 34]. We found that soybean trypsin inhibitor and antioxidants, NAC, DMTU and CDDOIm suppressed dust extract induction of NFκB-p65 phosphorylation (Fig. 9) suggesting that proteases and oxidant generation control inflammatory gene induction via activation of NF-κB.

### Protease inhibitors and antioxidants suppress activation of protein kinase C

**Fig. 10:**
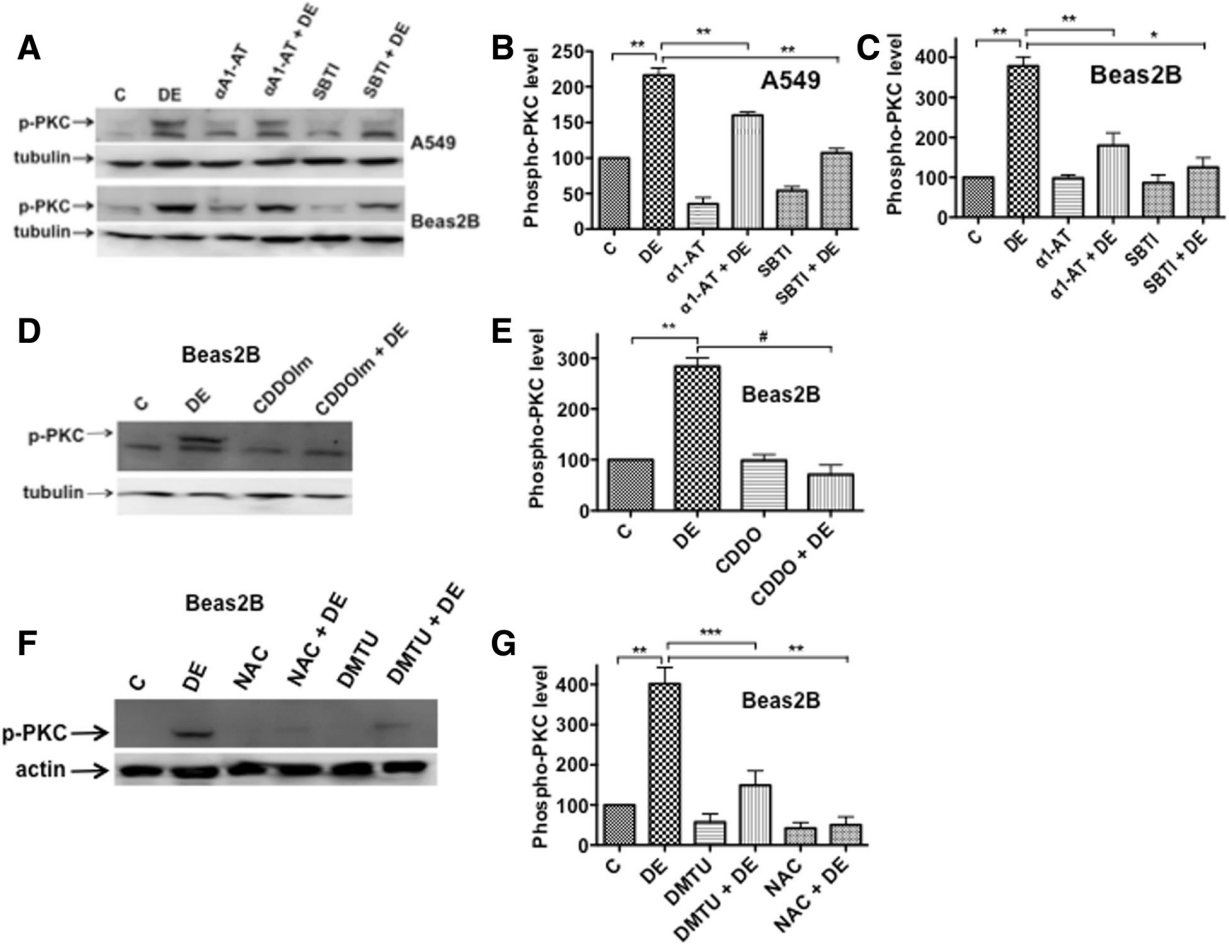
Effects of protease inhibitors and antioxidants on the activation of protein kinase C (PKC). **a** A549 or Beas2B cells were treated with medium (C), dust extract (1 %) (DE), α1-antitrypsin (25 μg/ml) (α1-AT) or soybean trypsin inhibitor (25 μg/ml) (SBTI) alone, or a combination of DE with α1-AT or SBTI for 5 min. A representative Western blot depicting the levels of phosphorylated PKC and tubulin is shown. **b** and **c** Quantification of the effects of α1-AT and SBTI on phosphorylated PKC levels. Levels of phosphorylated PKC and tubulin were quantified and phosphorylated PKC levels normalized to tubulin levels. Levels of phosphorylated PKC in cells treated with medium (C) were arbitrarily considered as 100, and data are shown as means ± SE (*n* = 3). *P < 0.05; **P < 0.005. **d** and **f** Beas2B cells were incubated first with medium (C), 0.5 μM CDDOIm or 15 mM n-acetylcysteine (NAC) adjusted to pH 7.0 for 3 h or 30 mM dimethylthioyrea (DMTU) for 1 h and then treated with or without 1 % dust extract for 5 min. Representative Western blots depicting the levels of phosphorylated PKC, tubulin and actin are shown. **e** and **g** Levels of phosphorylated PKC were normalized to tubulin or actin, and levels of phosphorylated PKC in cells treated with medium were arbitrarily considered as 100. Data shown are means ± SE (*n* = 3). **P < 0.01; ***P < 0.001; #*P* < 0.0001

We previously found that the activation of protein kinase C (PKC) signaling is necessary for poultry dust extract induction of IL-8 expression [14]. To determine if proteases in dust extracts and intracellular oxidants activate PKC, we analyzed the effects of α1-antitrypsin, soybean trypsin inhibitor or antioxidants, CDDOIm, NAC and DMTU on PKC activation by Western immunoblotting using an antibody against phospho-PKC (pan) that detects several isoforms of phosphorylated-PKC (Fig. 10). In agreement with our published studies [14], poultry dust extract rapidly increased PKC phosphorylation in A549 and Beas2b cells, and α1-antitrypsin, soybean trypsin inhibitor, CDDOIm, NAC and DMTU suppressed increase of PKC phosphorylation (Fig. 10).

## Discussion

Exposure to organic dust is a risk factor for the development of respiratory symptoms and respiratory diseases [2]. Respiratory symptoms and diseases are among the leading illness in agricultural workers [1]. Ongoing acute and chronic exposures to organic dust are associated with the development of bronchitis, asthma, pneumonitis, organic dust toxic syndrome and other respiratory diseases [1]. Despite the high incidence of respiratory symptoms and respiratory diseases in agricultural workers, our understanding of the underlying molecular mechanisms remains incomplete. Organic dust is a complex mixture of organic and inorganic components and contains significant levels of endotoxin [1]. Although organic dust is known to induce inflammatory cytokines [14, 35, 36] and inflammatory responses [37], the identities of responsible agents are not known and their mechanisms of action are not well understood. We previously found that poultry dust extracts induce IL-8 expression in lung epithelial and THP-1 cells primarily by increasing gene transcription and that activation of protein kinase C and mitogen activated protein kinases control induction of IL-8 [14]. Our data also demonstrated that dust extract induces AP-1 and NFκB DNA binding activities to increase IL-8 promoter activity [14]. Interestingly, we found that IL-8 induction was not sensitive to polymyxin B inhibition indicating that endotoxin present in dust extract may not act independently to induce IL-8 expression [14]. Our genome-wide expression profiling studies found that poultry dust extracts induce several chemokines, cytokines, and other inflammatory proteins in A549 and Beas2B lung epithelial and THP-1 cells [15].

In this study we report on the mechanisms by which protease activities in poultry dust extracts and intracellular oxidants induce inflammatory gene expression in lung epithelial cells. The presence of protease activities in organic dust and their role in the induction of inflammatory gene expression has not been thoroughly investigated. Previously, we detected elastase- and trypsin-like activities in aqueous extracts of poultry dust, and found that heat inactivation at 95 °C for 10 min and serine protease inhibitors such as, α1-antitrypsin and soybean trypsin inhibitor suppressed induction of IL-8 mRNA and protein levels in THP-1 monocytic cells (Bandari, S. K., Master’s Thesis, University of Texas Health Science Center at Tyler, 2012). While our studies were in progress, the presence of proteases in the swine barn dust and their effects on the induction of cytokine expression in lung cells and mouse lung via activation of PAR-1 and PAR-2 were reported [38]. A number of noteworthy points distinguish our studies from the recent report; our studies have independently provided further evidence for the involvement of protease activities in organic dust from an entirely different source, namely poultry, in the induction of inflammatory gene expression in lung epithelial cells. Furthermore, our studies demonstrate that proteases in poultry dust induce, in addition to IL-8 and IL-6, other genes implicated in lung inflammation such as IL-1β, CCL2, ICAM-1, PTGS2 and TLR4 in alveolar as well as bronchiolar epithelial cells. Our finding that proteases in poultry dust induce MMP-1, MMP-3, MMP-9 and MMP-13 mRNAs suggest that matrix metalloproteinases could be important regulators of organic dust induced lung inflammation. Our data on the associations between protease activities, intracellular oxidant generation and inflammatory gene induction raise the interesting question whether proteases are involved in the generation of intracellular oxidants. Our studies are the first to report on the involvement of oxidant stress in the organic dust induction of inflammatory gene expression in lung epithelial cells. Finally, our data demonstrated that proteases control IL-8 induction at the transcriptional level.

Measurement of protease activities and susceptibility to protease inhibitors indicated that dust extract contains trypsin- and elastase-like activities, however the true identities of the proteases and their sources remain to be determined. Microbial pathogens, mites, dander and animal secretions present in the CAFO environment could be potential sources of protease activities in dust. Heat inactivation and protease inhibitors significantly reduced IL-8 mRNA levels induced by dust extract indicating that protease activities are involved in the induction. Dust extract induction of IL-8 protein levels were significantly reduced by serine protease inhibitors such as, α1-antitrypsin, soybean trypsin inhibitor, leupeptin and aprotinin, but not by the cysteine protease inhibitor E64 indicating that the protease activities likely belong to the family of serine proteases. The lack of effect of metalloprotease inhibitor, GM60001 indicates that metalloproteases may not be major players in the induction of inflammatory gene expression. Inhibition with α1-antitrypsin significantly reduced dust extract induced expression of IL-6, IL-1β, CCL2, PTGS2, ICAM1 and TLR4 further indicating the involvement of protease activities in the induction of inflammatory gene expression. Dust extract induction of IL-8 promoter activity was suppressed by α1-antitrypsin indicating that transcriptional mechanisms are involved in the induction of IL-8 expression by proteases. It remains to be determined if transcriptional mechanisms also control induction of the other inflammatory genes. We found that α1-antitrypsin and soybean trypsin inhibitor suppressed increase of NF-κB p65 and PKC phosphorylation by dust extract. These data indicate that proteases in dust extracts activate protein kinase C and NF-κB to induce inflammatory gene expression.

We were particularly interested in the involvement of PAR-1 and PAR-2 in the induction of IL-8 and other inflammatory genes, as they have been implicated to play pro-inflammatory functions in the lung [24]. Increased expression of PAR-2 is associated with chronic lung conditions such as asthma [39], bronchitis [40] and bronchopulmonary dysplasia in preterm infants [41]. Dust extract treatment increased PAR-1 and PAR-2 cleavage in a time-dependent manner, which was sensitive to inhibition by protease inhibitors indicating that proteases specifically activate PAR-1 and PAR-2. The PAR-1-specific antagonist BMS200261 and siRNA knockdown of PAR-1 or PAR-2 significantly reduced induction of IL-8 expression by dust extract indicating that proteases activate PAR-1 and PAR-2 to induce IL-8 expression. Signaling pathways that control PAR-1 and PAR-2 mediated induction of IL-8 expression by dust extract are not known. We have previously demonstrated that dust extract activates protein kinase C and MAPK pathways to increase IL-8 expression, and increased AP-1 and NFκB binding to IL-8 promoter is necessary for induction of promoter activity [14]. PARs are a family of four cell surface G-protein coupled receptors that are activated by cleavage of an extracellular amino-terminal sequence that enables a tethered ligand to bind to the receptor and activate multiple signaling cascades [22]. PARs are mainly activated by serine proteases of endogenous and exogenous origin and are known to play important roles in tissue responses to injury and inflammation [22]. Although PARs have been well studied in the control of thrombosis and hemostasis [42], their roles in lung inflammation and injury have not been fully elucidated.

Lung epithelial cells express all the four PARs [23], and trypsin, elastase, and coagulation factors VIIa and Xa are known to serve as endogenous activators of PAR-1 and PAR-2 in the lung [22]. Activation of PARs results in phospholipase C activation and subsequent production of inositol triphosphate, Ca^2+^ mobilization and diacylglycerol activation of protein kinase C. PAR-1 and PAR-2 are also known to activate MAP kinase signaling cascades [22]. In A549, Beas2B lung epithelial cell lines and primary human bronchial epithelial cells (HBECs) activation of PAR-1 and/or PAR-2 increases expression inflammatory mediators such as IL-6, IL-8, prostaglandin E2 [23]. House dust mite allergens, Der p1, Der p3, and Der p9 activate PAR-1 and PAR-2 to induce IL-6 and IL-8 expression in lung epithelial cells [43, 44] indicating pro-inflammatory roles for PAR-1 and PAR-2 in the lung. A secreted protease from Pseudomonas aeruginosa [45] and proteases from cockroach [46] activate PAR-1 and PAR-2 to increase IL-6, IL-8 or KC and TNF-α expression via activation of AP-1 and NFκB. Mechanisms by which PARs regulate organic dust induction of inflammatory gene expression in lung epithelial cells remains to be elucidated.

Experiments in SAE cells showed similarities to A549 and Beas2B lung cell lines with regard to dust extract induction of inflammatory gene expression and inhibition by protease inhibitors and antioxidants. There were also differences, namely insensitivity to inhibition by n-acetylcysteine (NAC) and lack of induction of MMP-1, MMP-3 and MMP-9 mRNAs. These differences could stem from differences in their origin (alveolar/bronchial/small airway) and/or nature of the cells (malignant/transformed/primary). The responses of SAE cells could also be influenced by age, sex, race and other variables of the donor. Nevertheless, protease inhibitors and antioxidants dimethylthiourea and CDDOIm suppressed IL-8, CCL2, and ICAM-1 mRNA levels. The lack of effects of NAC could also suggest that these cells may not metabolize NAC.

The involvement of oxidant stress in the organic dust induction of inflammatory gene expression in lung epithelial cells has not been studied previously. We found that treatment of A549 and Beas2B cells with poultry dust extracts induced oxidative stress concomitant with induction of inflammatory gene expression. Antioxidants such as n-acetylcysteine, dimethylthiourea, and the Nrf2 activator CDDOIm suppressed the inductive effects of dust extracts indicating that oxidant stress controls inflammatory gene induction. Although all of the antioxidants tested suppressed induction of the majority of the genes, some of the effects varied among the antioxidants and between the cells. For example, although NAC and CDDOIm suppressed ICAM-1 expression in Beas2B cells, DMTU did not and while CDDOIm did not suppress TLR4 expression in A549 cells, it suppressed it in Beas2B cells. The differential effects of these agents may be related to the differences in their mode of action and the differences between the cell types. NAC can act as an antioxidant by directly scavenging free radicals and indirectly via increasing cellular reduced glutathione (GSH) levels. DMTU although best known for its effects as a scavenger of hydroxyl radicals, is also known to scavenge hypochlorous acid (HOCl) and inhibit Na^+^−Ca^+^ exchange [47]. The triterpenoid CDDOIm is a potent activator of Nrf2 transcription factor that upregulates the expression of a number of cytoprotective and antioxidant enzymes to maintain cellular redox homeostasis [48]. Oxidative stress has been implicated in the pathogenesis and exacerbation of lung diseases such as asthma, chronic obstructive pulmonary disease, acute respiratory distress syndrome and others [32]. Exposure of phagocytic cells to particulate matter such as asbestos and silica leads to the generation of increased production of reactive oxygen species [49]. Other than phagocytic cells, lung epithelial cells also undergo oxidative stress following exposure to diesel exhaust particle chemicals [50], infection with respiratory syncytial virus [51] or Streptococcus pneumonia [52].

**Fig. 11:**
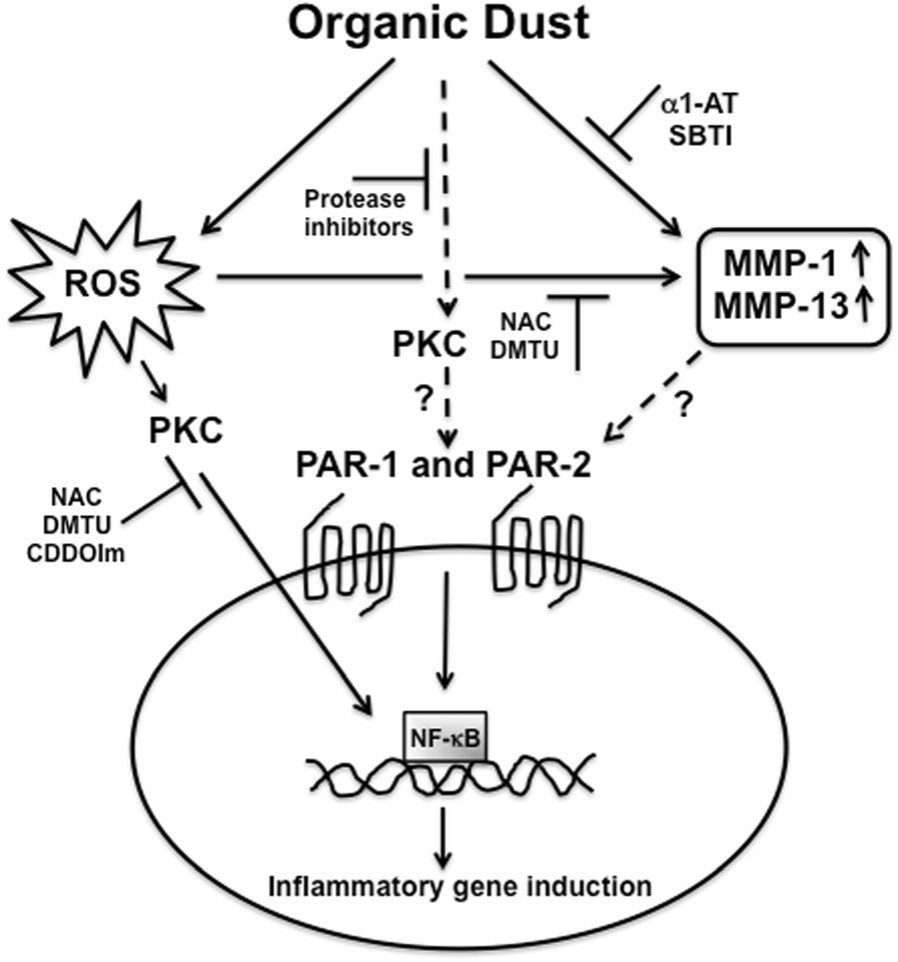
Mechanisms for the dust extract induction of inflammatory gene expression. The flow diagram shows how dust extracts induce inflammatory gene expression in lung epithelial cells. Exposure of cells to dust extracts increases intracellular reactive oxygen species (ROS) production and activates PAR-1 and PAR-2 to induce inflammatory gene expression. MMP-1 and MMP-13 that are induced by dust extract exposure can potentially activate PAR-1 to further modulate inflammatory gene expression. Protease inhibitors and antioxidants suppress protein kinase C (PKC) and NF-kB activation indicating that proteases and ROS are important players in signal transduction pathways for the induction of inflammatory gene expression. The role of PKC in the activation of PARs is yet to be studied. The inhibitors used to delineate the pathways are shown. α1-AT, alpha1-antitrypsin; SBTI, soybean trypsin inhibitor; NAC, n-acetylcysteine; DMTU, dimethylthiourea; CDDOIm, 1-(2-Cyano-3,12,28-trioxooleana-1,9(11)-dien-28-yl)-1H-imidazole. MMP, matrix metalloproteinase; PAR, protease activated receptor; PKC, protein kinase C, NF-kB, nuclear factor-kappaB

The production of cellular reactive oxygen species is mediated by the actions of enzymes such as NADPH oxidases, xanthine oxidase, and nitric oxide synthases or non-enzymatically by the mitochondrial electron transport chain [53]. The superoxide anion (O_2_^−^) formed is converted to hydrogen peroxide by superoxide dismutases. In the presence of ferrous or cuprous ions, hydrogen peroxide is converted into the highly reactive hydroxyl radical (^.^OH). The exact mechanisms for the production of ROS by dust extracts in our study are not known. Our data suggested that the oxidant-antioxidant imbalance in cells due to dust extract treatment leads to inflammatory gene induction. Transcription factors NF-κB and AP-1 that are activated by oxidative stress [54] control inflammatory cytokine and chemokine expression [31]. We also found previously that poultry dust extracts induce IL-8 expression in lung epithelial and THP-1 cells via activation of protein kinase signaling and NF-κB and AP-1 activation [14]. Antioxidants suppressed dust extract activation of protein kinase C and NF-κB indicating the involvement of oxidants. Together our data indicates that proteases and oxidants activate protein kinase C and NF-κB to induce inflammatory gene expression by dust extracts. The involvement of signal transduction pathways and the role of AP-1 and NF-κB in the protease and ROS mediated induction of inflammatory gene expression are yet to be studied in detail. The flow diagram summarizes our findings on the dust extract induction of inflammatory gene expression (Fig. 11).

Whether or not poultry dust extracts contain intrinsic oxidase activity such as NADPH oxidase activity and its role in the development of oxidative stress has not been investigated. Studies have demonstrated that ragweed [55] and birch pollen contain [56] NADPH oxidase activity and trigger ROS production when applied to the airway epithelium. Further, the intrinsic capacity of ragweed pollen to generate oxidative stress was found to amplify allergic airway inflammation. Our studies demonstrated that dust extracts contained serine protease-like activities and induce ROS production in A549 and Beas2B cells. It is not known if there is any link between protease activities in dust extracts and oxidative stress. Recently, it was demonstrated that intradermal injection of the cysteine protease papain resulted in elevated ROS production in skin epithelium and dermal dendritic cells in mice [57]. It will be interesting to determine if protease activities in poultry dust extracts induce ROS production thereby contributing to oxidative stress. Induction of inflammatory gene expression including expression of MMP mRNAs by dust extracts was sensitive to protease inhibitors and antioxidants. The induction of MMP-1 and MMP-13 is particularly interesting, as they are known to activate PAR-1 via cleavage at noncanonical sites that can contribute toward control of inflammatory gene expression.

## Conclusions

In summary, our studies have shown that poultry dust extracts induce inflammatory gene expression in lung epithelial cells via trypsin- and elastase-like activities and generation of oxidant stress. The protease activities activate PAR-1 and PAR-2 to induce IL-8 expression and likely other inflammatory genes. Proteases and oxidant stress activate PKC signaling and NF-κB to induce inflammatory gene expression. Signal transduction events subsequent to PKC activation, and gene regulatory mechanisms that control inflammatory gene induction remain to be elucidated. A better understanding of mechanisms underlying lung inflammatory responses to organic dust exposure will aid in the development of new interventions and treatments for acute and chronic lung diseases caused by organic dust exposure.

## Additional files

Additional file 1: Figure S1. Effects of protease inhibitors on IL-8 protein levels. A549 (A) and Beas2B (B–D) cells were treated with medium (C), 0.25 % dust extract (DE) or dust extract combined with 25 μg/ml α1-antitrypsin (α1-AT), soybean trypsin inhibitor (SBTI), 20 μM leupeptin, 5 μM aprotinin or 10 μM E64 for 3 h. IL-8 protein levels in cell medium and cell lysates were determined by ELISA. IL-8 protein levels in cell lysates were normalized to total protein content. IL-8 protein levels in dust extract treated medium or lysate were arbitrarily considered as 100 and relative levels in treated cells are shown. Data are shown as means ± SE (*n* = 3) for A549 cell lysate (A), means ± SD (n = 2) for Beas2B cell lysate and Beas2B medium (B and C), and means ± SE (*n* = 3) for Beas2B medium (D). (TIF 1520 kb)
Additional file 2: Figure S2. Effects of dust extract, antioxidants and protease inhibitors on cytotoxicity. A549 (A and B) and Beas2B (C and D) cells were incubated with medium (C), CDDOIm for 3 h or 30 mM dimethylthiourea (DMTU) for 1 h and then treated with or without 0.25 % dust extract (DE) for 3 h or treated with α1-antitrypsin (α1-AT) alone, soybean trypsin inhibitor (SBTI) alone, or in combination with 0.25 % dust extract for 3 h. Effects on cytotoxicity were determined by MTS assay by measuring optical density at 490 nm. Data shown are means ± SD of duplicate measurements. Similar results were obtained in a second independent experiment. (TIF 1520 kb)
Additional file 3: Figure S3. Effects of protease inhibitors and antioxidants by themselves on inflammatory gene mRNA levels in Beas2B cells. A and B. Cells were treated with medium (C), 10 μg/ml α1-antitrypsin (α1-AT), or 10 μg/ml soybean trypsin inhibitor (SBTI) for 3 h and the levels of inflammatory mRNAs were determined by qRT-PCR. The levels of mRNAs in cells treated with medium (C) were arbitrarily considered as 100. Data shown are means ± SD (*n* = 2). C–E. Cells were incubated with medium (C) or 30 mM dimethylthiourea (DMTU) for 4 h and 15 mM n-acetylcysteine (NAC) adjusted to pH 7.0, or 0.5 μM CDDOIm for 6 h and the levels of inflammatory mRNAs determined by qRT-PCR. Data shown are means ± SE (*n* = 3). **P* < 0.05; ***P* < 0.01, ****P* < 0.001 compared to cells treated with medium (C). (TIF 1520 kb)
Additional file 4: Figure S4. Effects of antioxidants on inflammatory gene expression. A549 (A and B) and Beas2B (C and D) cells were incubated first with medium (C) or medium containing 1 % dimethylsulfoxide (DMSO), 30 mM dimethylthiourea (DMTU), 30 mM Mannitol, or 5 mM diphenyleneiodonium (DPI) for 1 h, and 15 mM n-acetylcysteine (NAC) adjusted to pH 7.0 for 3 h and then treated with 0.25 % dust extract (DE) for 3 h. Levels of IL-8 mRNA and IL-8 protein in the medium were determined by qRT-PCR and ELISA, respectively. IL-8 mRNA and IL-8 protein levels in DE treated cells were arbitrarily considered as 100 and relative levels in other treatments are shown. Data are means ± SE (*n* = 3–6 for A549 and *n* = 3–4 for Beas2B). **P* < 0.05; ***P* < 0.01; ****P* < 0.001; #*P* < 0.0001. E. A549 cells were incubated first with medium (C) or 1 μM CDDOIm for 3 h and then treated with 0.25 % dust extract (DE) for 3 h. Levels of inflammatory mRNAs were determined by qRT-PCR. Level of each mRNA in dust extract treated cells was arbitrarily considered as 100 and relative levels in dust extract treated cells are shown. Data shown are means ± SD/SE (*n* = 2–3). (TIF 1520 kb)

## Abbreviations

4-HNE: 4-hydroxynonenal
AP: Alkaline phosphatase
BAPNA: Na-Benzoyl-D,L-arginine 4-nitroanilide hydrochloride
CAFOs: Concentrated animal feeding operations
CDDOIm: 1-(2-Cyano-3,12,28-trioxooleana-1,9(11)-dien-28-yl)-1*H*-imidazole
DHE: Dihydroethidium
DMSO: Dimethyl sulfoxide
DMTU: Dimethylthiourea
DPI: Diphenyleneiodonium
GCLC: Glutamate cysteine ligase catalytic subunit
GSH: Reduced glutathione
HBECs: Human bronchial epithelial cells
HMOX-1: Heme oxygenase-1
HOCl: Hypochlorous acid
MMP: Matrix metalloproteinase
MOI: Multiplicity of infection
NAC: N-acetylcysteine
NF-κB: Nuclear factor kappa B
NQO1: NADPH quinone oxidoreductase 1
PAR: Protease activated receptor
PKC: Protein kinase C
PMA: Phorbol myristate acetate
ROS: Reactive oxygen species
SAPNA: N-Suc-Ala-Ala-Ala-pNA
SBTI: Soybean trypsin inhibitor
α1-AT: alpha1-antitrypsin

## References

[1] Respiratory health hazards in agriculture. Am J Respir Crit Care Med. 1998;158(5 Pt 2):S1–76. 10.1164/ajrccm.158.supplement_1.rccm1585s1 9817727

[2] Kirkhorn SR, Garry VF (2000). Agricultural lung diseases. Environ Health Perspect.

[3] Heederik D, Sigsgaard T, Thorne PS, Kline JN, Avery R, Bonlokke JH, Chrischilles EA, Dosman JA, Duchaine C, Kirkhorn SR (2007). Health effects of airborne exposures from concentrated animal feeding operations. Environ Health Perspect.

[4] Larsson KA, Eklund AG, Hansson LO, Isaksson BM, Malmberg PO (1994). Swine dust causes intense airways inflammation in healthy subjects. Am J Respir Crit Care Med.

[5] Larsson BM, Palmberg L, Malmberg PO, Larsson K (1997). Effect of exposure to swine dust on levels of IL-8 in airway lavage fluid. Thorax.

[6] Radon K, Weber C, Iversen M, Danuser B, Pedersen S, Nowak D (2001). Exposure assessment and lung function in pig and poultry farmers. Occup Environ Med.

[7] Simpson JC, Niven RM, Pickering CA, Fletcher AM, Oldham LA, Francis HM (1998). Prevalence and predictors of work related respiratory symptoms in workers exposed to organic dusts. Occup Environ Med.

[8] Kirychuk SP, Senthilselvan A, Dosman JA, Juorio V, Feddes JJ, Willson P, Classen H, Reynolds SJ, Guenter W, Hurst TS (2003). Respiratory symptoms and lung function in poultry confinement workers in Western Canada. Can Respir J.

[9] Larsson BM, Larsson K, Malmberg P, Martensson L, Palmberg L (1999). Airway responses in naive subjects to exposure in poultry houses: comparison between cage rearing system and alternative rearing system for laying hens. Am J Ind Med.

[10] Viegas S, Faisca VM, Dias H, Clerigo A, Carolino E, Viegas C (2013). Occupational exposure to poultry dust and effects on the respiratory system in workers. J Toxicol Environ Health A.

[11] Shaykhiev R, Bals R (2007). Interactions between epithelial cells and leukocytes in immunity and tissue homeostasis. J Leukoc Biol.

[12] Parker D, Prince A (2011). Innate immunity in the respiratory epithelium. Am J Respir Cell Mol Biol.

[13] Chuquimia OD, Petursdottir DH, Periolo N, Fernandez C (2013). Alveolar epithelial cells are critical in protection of the respiratory tract by secretion of factors able to modulate the activity of pulmonary macrophages and directly control bacterial growth. Infect Immun.

[14] Gottipati KR, Bandari SK, Nonnenmann MW, Levin JL, Dooley GP, Reynolds SJ, Boggaram V (2015). Transcriptional mechanisms and protein kinase signaling mediate organic dust induction of IL-8 expression in lung epithelial and THP-1 cells. Am J Physiol Lung Cell Mol Physiol.

[15] Boggaram V, Loose DS, Gottipati KR, Natarajan K, Mitchell CT (2016). Gene expression profiling of the effects of organic dust in lung epithelial and THP-1 cells reveals inductive effects on inflammatory and immune response genes. Physiol Genomics.

[16] Awasthi V, Mandal SK, Papanna V, Rao LV, Pendurthi UR (2007). Modulation of tissue factor-factor VIIa signaling by lipid rafts and caveolae. Arterioscler Thromb Vasc Biol.

[17] Sen P, Gopalakrishnan R, Kothari H, Keshava S, Clark CA, Esmon CT, Pendurthi UR, Rao LV (2011). Factor VIIa bound to endothelial cell protein C receptor activates protease activated receptor-1 and mediates cell signaling and barrier protection. Blood.

[18] Churg A, Wang X, Wang RD, Meixner SC, Pryzdial EL, Wright JL (2007). Alpha1-antitrypsin suppresses TNF-alpha and MMP-12 production by cigarette smoke-stimulated macrophages. Am J Respir Cell Mol Biol.

[19] Sarin A, Nakajima H, Henkart PA (1995). A protease-dependent TCR-induced death pathway in mature lymphocytes. J Immunol.

[20] Lu Z, Korotcova L, Murata A, Ishibashi N, Jonas RA (2014). Aprotinin, but not epsilon-aminocaproic acid and tranexamic acid, exerts neuroprotection against excitotoxic injury in an in vitro neuronal cell culture model. J Thorac Cardiovasc Surg.

[21] Post S, Nawijn MC, Hackett TL, Baranowska M, Gras R, van Oosterhout AJ, Heijink IH (2012). The composition of house dust mite is critical for mucosal barrier dysfunction and allergic sensitisation. Thorax.

[22] Ossovskaya VS, Bunnett NW (2004). Protease-activated receptors: contribution to physiology and disease. Physiol Rev.

[23] Asokananthan N, Graham PT, Fink J, Knight DA, Bakker AJ, McWilliam AS, Thompson PJ, Stewart GA (2002). Activation of protease-activated receptor (PAR)-1, PAR-2, and PAR-4 stimulates IL-6, IL-8, and prostaglandin E2 release from human respiratory epithelial cells. J Immunol.

[24] Kawabata A, Kawao N (2005). Physiology and pathophysiology of proteinase-activated receptors (PARs): PARs in the respiratory system: cellular signaling and physiological/pathological roles. J Pharmacol Sci.

[25] Quinton TM, Kim S, Derian CK, Jin J, Kunapuli SP (2004). Plasmin-mediated activation of platelets occurs by cleavage of protease-activated receptor 4. J Biol Chem.

[26] Chakraborti S, Mandal M, Das S, Mandal A, Chakraborti T (2003). Regulation of matrix metalloproteinases: an overview. Mol Cell Biochem.

[27] Van Lint P, Libert C (2007). Chemokine and cytokine processing by matrix metalloproteinases and its effect on leukocyte migration and inflammation. J Leukoc Biol.

[28] Trivedi V, Boire A, Tchernychev B, Kaneider NC, Leger AJ, O’Callaghan K, Covic L, Kuliopulos A (2009). Platelet matrix metalloprotease-1 mediates thrombogenesis by activating PAR1 at a cryptic ligand site. Cell.

[29] Jaffre F, Friedman AE, Hu Z, Mackman N, Blaxall BC (2012). Beta-adrenergic receptor stimulation transactivates protease-activated receptor 1 via matrix metalloproteinase 13 in cardiac cells. Circulation.

[30] Austin KM, Covic L, Kuliopulos A (2013). Matrix metalloproteases and PAR1 activation. Blood.

[31] Reuter S, Gupta SC, Chaturvedi MM, Aggarwal BB (2010). Oxidative stress, inflammation, and cancer: how are they linked?. Free Radic Biol Med.

[32] Ciencewicki J, Trivedi S, Kleeberger SR (2008). Oxidants and the pathogenesis of lung diseases. J Allergy Clin Immunol.

[33] Sasaki CY, Barberi TJ, Ghosh P, Longo DL (2005). Phosphorylation of RelA/p65 on serine 536 defines an I{kappa}B{alpha}-independent NF-{kappa}B pathway. J Biol Chem.

[34] Christian F, Smith EL, Carmody RJ (2016). The Regulation of NF-kappaB Subunits by Phosphorylation. Cells.

[35] Romberger DJ, Bodlak V, Von Essen SG, Mathisen T, Wyatt TA (2002). Hog barn dust extract stimulates IL-8 and IL-6 release in human bronchial epithelial cells via PKC activation. J Appl Physiol.

[36] Wyatt TA, Slager RE, Devasure J, Auvermann BW, Mulhern ML, Von Essen S, Mathisen T, Floreani AA, Romberger DJ (2007). Feedlot dust stimulation of interleukin-6 and -8 requires protein kinase Cepsilon in human bronchial epithelial cells. Am J Physiol Lung Cell Mol Physiol.

[37] Poole JA, Wyatt TA, Oldenburg PJ, Elliott MK, West WW, Sisson JH, Von Essen SG, Romberger DJ (2009). Intranasal organic dust exposure-induced airway adaptation response marked by persistent lung inflammation and pathology in mice. Am J Physiol Lung Cell Mol Physiol.

[38] Romberger DJ, Heires AJ, Nordgren TM, Souder CP, West W, Liu XD, Poole JA, Toews ML, Wyatt TA (2015). Proteases in agricultural dust induce lung inflammation through PAR-1 and PAR-2 activation. Am J Physiol Lung Cell Mol Physiol.

[39] Knight DA, Lim S, Scaffidi AK, Roche N, Chung KF, Stewart GA, Thompson PJ (2001). Protease-activated receptors in human airways: upregulation of PAR-2 in respiratory epithelium from patients with asthma. J Allergy Clin Immunol.

[40] Miotto D, Hollenberg MD, Bunnett NW, Papi A, Braccioni F, Boschetto P, Rea F, Zuin A, Geppetti P, Saetta M (2002). Expression of protease activated receptor-2 (PAR-2) in central airways of smokers and non-smokers. Thorax.

[41] Cederqvist K, Haglund C, Heikkila P, Hollenberg MD, Karikoski R, Andersson S (2005). High expression of pulmonary proteinase-activated receptor 2 in acute and chronic lung injury in preterm infants. Pediatr Res.

[42] Coughlin SR (2005). Protease-activated receptors in hemostasis, thrombosis and vascular biology. J Thromb Haemost.

[43] Sun G, Stacey MA, Schmidt M, Mori L, Mattoli S (2001). Interaction of mite allergens Der p3 and Der p9 with protease-activated receptor-2 expressed by lung epithelial cells. J Immunol.

[44] Asokananthan N, Graham PT, Stewart DJ, Bakker AJ, Eidne KA, Thompson PJ, Stewart GA (2002). House dust mite allergens induce proinflammatory cytokines from respiratory epithelial cells: the cysteine protease allergen, Der p 1, activates protease-activated receptor (PAR)-2 and inactivates PAR-1. J Immunol.

[45] Kida Y, Higashimoto Y, Inoue H, Shimizu T, Kuwano K (2008). A novel secreted protease from Pseudomonas aeruginosa activates NF-kappaB through protease-activated receptors. Cell Microbiol.

[46] Day SB, Zhou P, Ledford JR, Page K (2010). German cockroach frass proteases modulate the innate immune response via activation of protease-activated receptor-2. J Innate Immun.

[47] Wasil M, Halliwell B, Grootveld M, Moorhouse CP, Hutchison DC, Baum H (1987). The specificity of thiourea, dimethylthiourea and dimethyl sulphoxide as scavengers of hydroxyl radicals. Their protection of alpha 1-antiproteinase against inactivation by hypochlorous acid. Biochem J.

[48] Thimmulappa RK, Fuchs RJ, Malhotra D, Scollick C, Traore K, Bream JH, Trush MA, Liby KT, Sporn MB, Kensler TW (2007). Preclinical evaluation of targeting the Nrf2 pathway by triterpenoids (CDDO-Im and CDDO-Me) for protection from LPS-induced inflammatory response and reactive oxygen species in human peripheral blood mononuclear cells and neutrophils. Antioxid Redox Signal.

[49] Vallyathan V, Shi X, Castranova V (1998). Reactive oxygen species: their relation to pneumoconiosis and carcinogenesis. Environ Health Perspect.

[50] Li N, Wang M, Oberley TD, Sempf JM, Nel AE (2002). Comparison of the pro-oxidative and proinflammatory effects of organic diesel exhaust particle chemicals in bronchial epithelial cells and macrophages. J Immunol.

[51] Hosakote YM, Liu T, Castro SM, Garofalo RP, Casola A (2009). Respiratory syncytial virus induces oxidative stress by modulating antioxidant enzymes. Am J Respir Cell Mol Biol.

[52] Zahlten J, Kim YJ, Doehn JM, Pribyl T, Hocke AC, Garcia P, Hammerschmidt S, Suttorp N, Hippenstiel S, Hubner RH (2015). Streptococcus pneumoniae-Induced Oxidative Stress in Lung Epithelial Cells Depends on Pneumococcal Autolysis and Is Reversible by Resveratrol. J Infect Dis.

[53] Droge W (2002). Free radicals in the physiological control of cell function. Physiol Rev.

[54] Pinkus R, Weiner LM, Daniel V (1996). Role of oxidants and antioxidants in the induction of AP-1, NF-kappaB, and glutathione S-transferase gene expression. J Biol Chem.

[55] Boldogh I, Bacsi A, Choudhury BK, Dharajiya N, Alam R, Hazra TK, Mitra S, Goldblum RM, Sur S (2005). ROS generated by pollen NADPH oxidase provide a signal that augments antigen-induced allergic airway inflammation. J Clin Invest.

[56] Shalaby KH, Allard-Coutu A, O’Sullivan MJ, Nakada E, Qureshi ST, Day BJ, Martin JG (2013). Inhaled birch pollen extract induces airway hyperresponsiveness via oxidative stress but independently of pollen-intrinsic NADPH oxidase activity, or the TLR4-TRIF pathway. J Immunol.

[57] Tang H, Cao W, Kasturi SP, Ravindran R, Nakaya HI, Kundu K, Murthy N, Kepler TB, Malissen B, Pulendran B (2010). The T helper type 2 response to cysteine proteases requires dendritic cell-basophil cooperation via ROS-mediated signaling. Nat Immunol.

