# Supplementary figures and images for: Proteases and oxidant stress control organic dust induction of inflammatory gene expression in lung epithelial cells
Source: Respir Res. 2016 Oct 22;17:137. doi: 10.1186/s12931-016-0455-z (PMC5075176; doi:10.1186/s12931-016-0455-z)

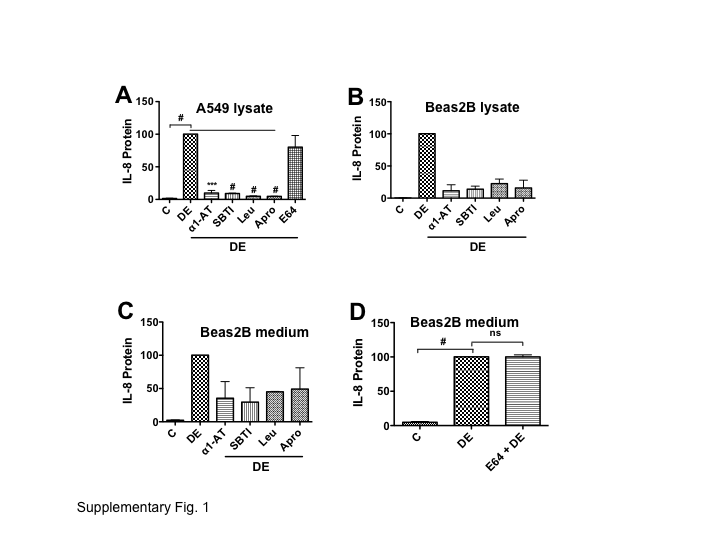

Supplement: Additional file 1: Figure S1. — Effects of protease inhibitors on IL-8 protein levels. A549 (A) and Beas2B (B–D) cells were treated with medium (C), 0.25 % dust extract (DE) or dust extract combined with 25 μg/ml α1-antitrypsin (α1-AT), soybean trypsin inhibitor (SBTI), 20 μM leupeptin, 5 μM aprotinin or 10 μM E64 for 3 h. IL-8 protein levels in cell medium and cell lysates were determined by ELISA. IL-8 protein levels in cell lysates were normalized to total protein content. IL-8 protein levels in dust extract treated medium or lysate were arbitrarily considered as 100 and relative levels in treated cells are shown. Data are shown as means ± SE (n = 3) for A549 cell lysate (A), means ± SD (n = 2) for Beas2B cell lysate and Beas2B medium (B and C), and means ± SE (n = 3) for Beas2B medium (D). (TIF 1520 kb) [file 12931_2016_455_MOESM1_ESM.tif]

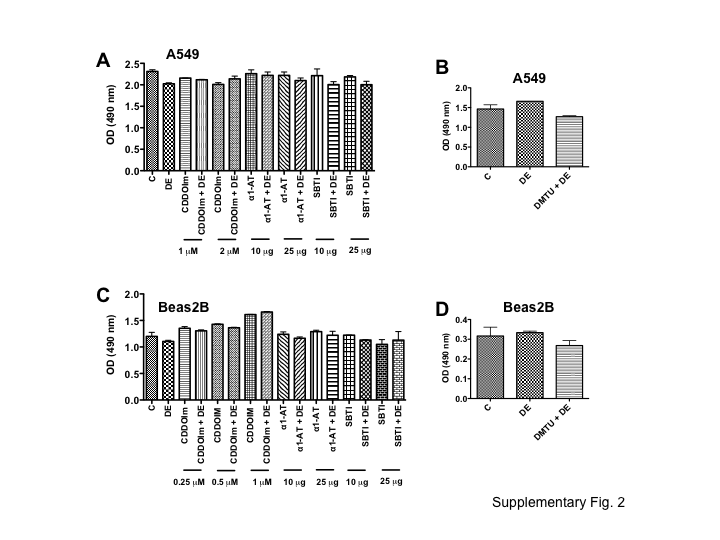

Supplement: Additional file 2: Figure S2. — Effects of dust extract, antioxidants and protease inhibitors on cytotoxicity. A549 (A and B) and Beas2B (C and D) cells were incubated with medium (C), CDDOIm for 3 h or 30 mM dimethylthiourea (DMTU) for 1 h and then treated with or without 0.25 % dust extract (DE) for 3 h or treated with α1-antitrypsin (α1-AT) alone, soybean trypsin inhibitor (SBTI) alone, or in combination with 0.25 % dust extract for 3 h. Effects on cytotoxicity were determined by MTS assay by measuring optical density at 490 nm. Data shown are means ± SD of duplicate measurements. Similar results were obtained in a second independent experiment. (TIF 1520 kb) [file 12931_2016_455_MOESM2_ESM.tif]

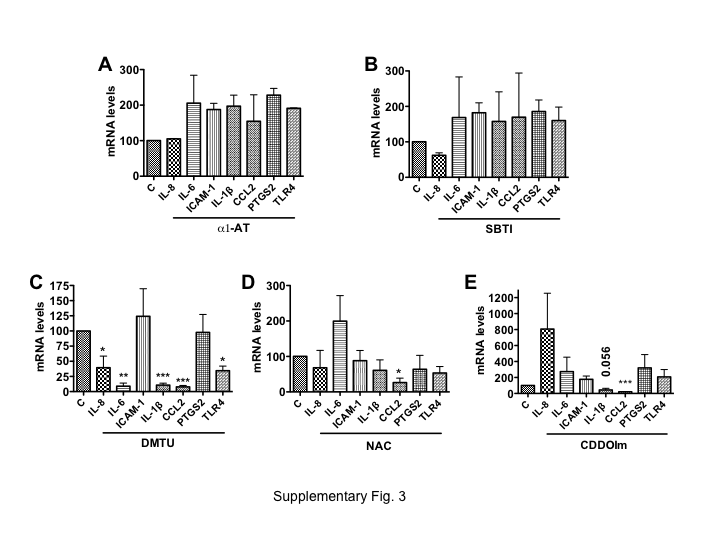

Supplement: Additional file 3: Figure S3. — Effects of protease inhibitors and antioxidants by themselves on inflammatory gene mRNA levels in Beas2B cells. A and B. Cells were treated with medium (C), 10 μg/ml α1-antitrypsin (α1-AT), or 10 μg/ml soybean trypsin inhibitor (SBTI) for 3 h and the levels of inflammatory mRNAs were determined by qRT-PCR. The levels of mRNAs in cells treated with medium (C) were arbitrarily considered as 100. Data shown are means ± SD (n = 2). C–E. Cells were incubated with medium (C) or 30 mM dimethylthiourea (DMTU) for 4 h and 15 mM n-acetylcysteine (NAC) adjusted to pH 7.0, or 0.5 μM CDDOIm for 6 h and the levels of inflammatory mRNAs determined by qRT-PCR. Data shown are means ± SE (n = 3). *P < 0.05; **P < 0.01, ***P < 0.001 compared to cells treated with medium (C). (TIF 1520 kb) [file 12931_2016_455_MOESM3_ESM.tif]

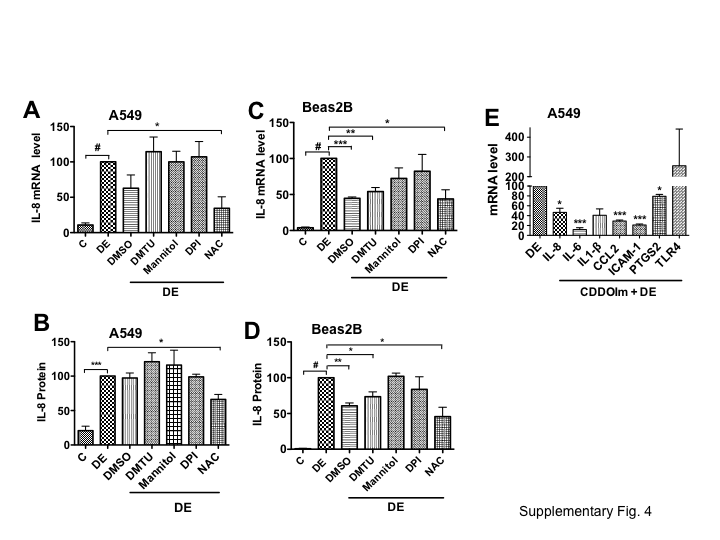

Supplement: Additional file 4: Figure S4. — Effects of antioxidants on inflammatory gene expression. A549 (A and B) and Beas2B (C and D) cells were incubated first with medium (C) or medium containing 1 % dimethylsulfoxide (DMSO), 30 mM dimethylthiourea (DMTU), 30 mM Mannitol, or 5 mM diphenyleneiodonium (DPI) for 1 h, and 15 mM n-acetylcysteine (NAC) adjusted to pH 7.0 for 3 h and then treated with 0.25 % dust extract (DE) for 3 h. Levels of IL-8 mRNA and IL-8 protein in the medium were determined by qRT-PCR and ELISA, respectively. IL-8 mRNA and IL-8 protein levels in DE treated cells were arbitrarily considered as 100 and relative levels in other treatments are shown. Data are means ± SE (n = 3–6 for A549 and n = 3–4 for Beas2B). *P < 0.05; **P < 0.01; ***P < 0.001; #P < 0.0001. E. A549 cells were incubated first with medium (C) or 1 μM CDDOIm for 3 h and then treated with 0.25 % dust extract (DE) for 3 h. Levels of inflammatory mRNAs were determined by qRT-PCR. Level of each mRNA in dust extract treated cells was arbitrarily considered as 100 and relative levels in dust extract treated cells are shown. Data shown are means ± SD/SE (n = 2–3). (TIF 1520 kb) [file 12931_2016_455_MOESM4_ESM.tif]
